# Synchronous Chaos and Broad Band Gamma Rhythm in a Minimal Multi-Layer Model of Primary Visual Cortex

**DOI:** 10.1371/journal.pcbi.1002176

**Published:** 2011-10-06

**Authors:** Demian Battaglia, David Hansel

**Affiliations:** 1 Max Planck Institute for Dynamics and Self-Organization, Göttingen, Germany; 2 Bernstein Center for Computational Neuroscience, Göttingen, Germany; 3 Université Paris Descartes, Laboratoire de Neurophysique et Physiologie, CNRS UMR 8119, Paris, France; 4 Interdisciplinary Center for Neural Computation, The Hebrew University, Jerusalem, Israel; Indiana University, United States of America

## Abstract

Visually induced neuronal activity in V1 displays a marked gamma-band component which is modulated by stimulus properties. It has been argued that synchronized oscillations contribute to these gamma-band activity. However, analysis of Local Field Potentials (LFPs) across different experiments reveals considerable diversity in the degree of oscillatory behavior of this induced activity. Contrast-dependent power enhancements can indeed occur over a broad band in the gamma frequency range and spectral peaks may not arise at all. Furthermore, even when oscillations are observed, they undergo temporal decorrelation over very few cycles. This is not easily accounted for in previous network modeling of gamma oscillations. We argue here that interactions between cortical layers can be responsible for this fast decorrelation. We study a model of a V1 hypercolumn, embedding a simplified description of the multi-layered structure of the cortex. When the stimulus contrast is low, the induced activity is only weakly synchronous and the network resonates transiently without developing collective oscillations. When the contrast is high, on the other hand, the induced activity undergoes synchronous oscillations with an irregular spatiotemporal structure expressing a synchronous chaotic state. As a consequence the population activity undergoes fast temporal decorrelation, with concomitant rapid damping of the oscillations in LFPs autocorrelograms and peak broadening in LFPs power spectra. We show that the strength of the inter-layer coupling crucially affects this spatiotemporal structure. We predict that layer VI inactivation should induce global changes in the spectral properties of induced LFPs, reflecting their slower temporal decorrelation in the absence of inter-layer feedback. Finally, we argue that the mechanism underlying the emergence of synchronous chaos in our model is in fact very general. It stems from the fact that gamma oscillations induced by local delayed inhibition tend to develop chaos when coupled by sufficiently strong excitation.

## Author Summary

Visual stimulation elicits neuronal responses in visual cortex. When the contrast of the used stimuli increases, the power of this induced activity is boosted over a broad frequency range (30–100 Hz), called the “gamma band.” It would be tempting to hypothesize that this phenomenon is due to the emergence of oscillations in which many neurons fire collectively in a rhythmic way. However, previous models trying to explain contrast-related power enhancements using synchronous oscillations failed to reproduce the observed spectra because they originated unrealistically sharp spectral peaks. The aim of our study is to reconcile synchronous oscillations with broad-band power spectra. We argue here that, thanks to the interaction between neuronal populations at different depths in the cortical tissue, the induced oscillatory responses are synchronous, but, at the same time, chaotic. The chaotic nature of the dynamics makes it possible to have broad-band power spectra together with synchrony. Our modeling study allows us formulating qualitative experimental predictions that provide a potential test for our theory. We predict that if the interactions between cortical layers are suppressed, for instance by inactivating neurons in deep layers, the induced responses might become more regular and narrow isolated peaks might develop in their power spectra.

## Introduction

An increase of activity in the gamma band (30–100 Hz) is observed in Local Field Potential (LFP) and Multi-Unit Activity (MUA) recordings [1]–[14], as well as in EEG and Electrocorticogram studies [15], [16] in primary visual cortex (V1) upon visual stimulation. Gamma activity is modulated by properties of the presented stimulus, such as orientation [2], [14], [17], contrast [7], [9], [18], velocity [3], [4] or size [12], much more strongly than the change in power in other frequency bands [19], [20]. Local GABA-ergic interneuronal networks are thought to play a key role in the production of neuronal activity in the gamma range ([21], see [22] for a review), as upheld as well by recent results obtained through optogenetic techniques in-vivo [23], [24].

Modeling works have provided a theoretical basis to account for the way in which networks of inhibitory interneurons can generate synchronous oscillatory activity in the gamma range [25]–[30]. In brief, in one possible scenario, the dynamics of the inhibitory post-synaptic currents is non-instantaneous (due to axonal delays, but also simply to finite synaptic time-constants). This contributes to create narrow time-windows in which excitatory and inhibitory neurons can fire closely in-phase, before being prevented to do so by a delayed inhibitory feedback. Therefore delayed inhibition, without need of an active involvement of excitatory populations, is capable inducing collective synchronous oscillations in neuronal activity. The frequency of these oscillations falls in the gamma band if the synaptic time constant of the inhibition is in an appropriate range. If a network operates in such a synchronous regime the neurons are engaged into approximately periodic collective oscillations involving a macroscopically large number of neurons. Therefore these oscillations are weakly affected by local noise and they maintain coherence over arbitrarily long time intervals. Power spectra of population observables of the network activity (e.g. LFP or MUA) exhibit narrow harmonic-like peaks and the damping of the corresponding autocorrelograms is slow.

Peaks in the gamma-band have been identified in the LFP or MUA spectra of induced activity in-vivo in V1 [1]–[4], [12]. However, in general these peaks are very broad and in many cases they are virtually indistinguishable as the stimulus-modulated gamma power of the signals spreads across a broad-band frequency interval [7], [9], [10], [31]–[33]. Characterization of the spatio-temporal structure of the gamma induced activity by means of auto-correlations (AC) and cross-correlations (CC) of single-unit, multi-unit and LFP signals has also revealed that the neuronal activity has a tendency to oscillate, which can be stronger or weaker, depending on the considered experiment. In some cases the oscillatory components of ACs and CCs of the induced activity display many cycles before getting damped [1], [2], [12], [14]. In other cases, however, the oscillations are completely damped after one or two cycles [3], [8], [13], [17]. The existence of different dynamical regimes might underlie this observed diversity.

For the mathematical abstraction of infinitely large networks, sharp boundaries between asynchronous and synchronous dynamical states exist [34], but for networks of a finite size such transitions are fuzzier [25], [34], [35]. Consequently, if the network does not operate too far from the instability to collective oscillations, in a regime which is formally defined as asynchronous –see [34], [35] and below for the definition–, the dominant normal modes of the network, which describe its response to small perturbations, can display damped oscillations at gamma frequencies. Local noise can excite these modes, inducing short-lived episodes of synchronous oscillatory activity. However, since these episodes are transient, the subsequent increase in power at gamma frequencies is broad-band. Induced broad band gamma power increases in V1 can therefore be accounted for if one assumes that the V1 network operates in such an asynchronous regime at the edge of developing synchrony [36]–[38]. In this regime, correlations in the spikes as well as in the membrane potentials of pairs of neurons are in general weak unless the neurons are connected via strong and direct synapses. However, in order to get a significant, although damped, oscillatory component in the macroscopic activity, the network must be “at the edge of synchronization”. In models, parameters have to be tuned in such a way to be close to an instability toward fully-developed synchronous oscillations, and this tuning have to be tighter, the larger the size of the recruited network [25], [29]. It is not clear how the required fine tuning would be satisfied given the range of experimental conditions in which gamma oscillations have been observed.

In the present study we explore another scenario which reconciles collective synchronous activity with broad-band spectral modulations and robust fast decoherence. It is based on a mechanism proposed recently for the emergence of synchronous chaos in recurrent neural networks [39]–[41]. In this mechanism, clusters of neuron undergo a synchronous gamma oscillation due to local mutual inhibition. These collective gamma oscillations become chaotic when the neuronal clusters are allowed to interact through longer-range excitation. The resulting overall patterns of activity are characterized by synchrony at the population level, but at a same time display a characteristic lack of temporal regularity due to chaos. As a consequence, the power of this activity spreads over a broad interval of frequencies and the oscillatory components of the autocorrelograms of neuronal activity and LFP signals are rapidly damped within a few tenths of a millisecond. In this alternative regime, correlations in the spikes of pairs of neurons are still weak and go together with the sparseness of the firing, but correlations in their membrane potentials can be strong.

We present here a model of a hypercolumn in V1, endowed with a simplified multi-layer architecture. In order to explain broad-band contrast-dependent spectral modulations in terms of synchronous chaos, we need to identify distinct interacting oscillators within the local cortical circuit. We hypothesize that neuronal populations within different thalamo-recipient cortical layers are set into oscillation by increased driving and that the mutual interaction between these populations, mediated by inter-layer synaptic connections, supports the development of synchronous chaos. This hypothesis is backed up by anatomical evidence. Thalamo-cortical synapses, providing direct sensory-induced driving, indeed target cortical layer IV but also, to a lesser extent, layer VI [42]–[46]. Extensive networks of recurrent inhibitory connections are present within each thalamo-recipient layer [45], [47], [48], supporting local generation of oscillations at multiple depths in the cortical tissue. Finally, stereotyped circuit motifs provide a bidirectional poly-synaptic connection loop between thalamo-recipient layers [44]–[46], [49]–[51].

Relying on extensive numerical simulations, we show that our model displays broad-band gamma modulations of the spectra of LFPs upon stimulation of the network at low as well as at high contrast. Whereas this induced activity is asynchronous at low contrast, it develops synchrony on a macroscopic scale when the contrast increases. Therefore we argue that the broad band gamma power observed in recorded LFP spectra in V1 is compatible with the existence of visually induced synchronous oscillatory neuronal dynamics.

## Results

### Multi-layer hypercolumn model

**Figure 1:**
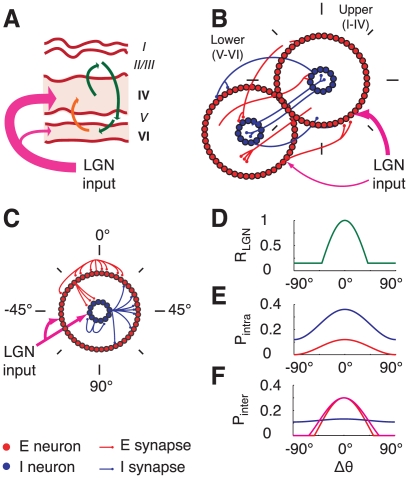
Schematic drawing of the model hypercolumns. A: cartoon of the loop circuit among the 6 layers of striate cortex. Thalamo-recipient layers are indicated by pink shading. B: two-rings network, corresponding to a hypercolumn with interacting layers. LGN inputs are weaker toward the lower layer than toward the upper layer. C: the single ring network for each layer of the model hypercolumn. LGN inputs target both excitatory and inhibitory neurons. D: spatial profile of LGN input. E: spatial modulation of the probability of connections between two cells in the same layer, separated by an angular distance 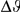. Red line: excitatory connections. Blue line: inhibitory connections. F: spatial modulation of the probability of connections between two cells in different layers, separated by an angular distance 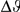. Red line: upper-to-lower layer excitatory connections and lower-to-upper excitatory connections toward excitatory neurons. Magenta line: lower-to-upper layer excitatory connections toward inhibitory neurons. Blue line: lower-to-upper and upper-to-lower layer inhibitory connections.

We model a functional hypercolumn in primary visual cortex as a large recurrent network of spiking integrate-and-fire-type neurons. To account in a simplified way for the layered structure of the visual cortex –a cartoon of which is shown in Figure 1A– the model network consists of two sub-networks, schematically representing layers I to IV and layers V to VI. We denote these two sub-networks as the *upper* and *lower layer* respectively (Figure 1B). Each of these layers comprises 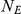 excitatory and 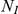 inhibitory neurons, for a total number of 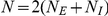 neurons in the network. Most of the simulations in this study are performed taking 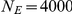 excitatory and 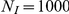 inhibitory neurons per layer, leading a total of 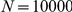 neurons in the model hypercolumn. This number is one order of magnitude smaller than estimates of the number of neurons in a real V1 hypercolumn based on neuronal densities recently measured by [52]. However, it leads to dynamical behaviors similar to larger network sizes (see following scaling analyses) and constitutes a compromise for efficient and fast simulations.

Each layer is described by a network with the geometry of a ring as depicted in Figure 1C, with neurons labeled by angular coordinates, 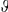, ranging from −90 to +90 degrees [53], [54]. The connections between neurons within each layer are random, with connection probabilities that depend on the angular distance between pre- and post-synaptic neurons. Spatial averages and spatial modulations of connection probabilities are set independently for the various kinds of connections (e.g. excitatory-to-excitatory, excitatory-to-inhibitory, inhibitory-to-excitatory or inhibitory-to-inhibitory), thus making it possible to vary the spatial profiles of net synaptic interactions (see Figure 1D, E, F). Excitatory and inhibitory inter-layer connections are also random and spatially modulated. All the external inputs to the network are modeled as stochastic processes (see Methods section). The neurons receive an external non-selective noisy current representing background inputs to V1 from other brain areas and a weakly tuned noisy current which represents visually induced inputs to V1 from converging Lateral Geniculate Nucleus (LGN) synapses [55]. Note that the two main thalamo-recipient layers, i.e. layers VI and IV, are embedded within two distinct model layers.

Our two-layer circuit embeds in a simplified manner several known features of the stereotypical interlaminar anatomy of the columnar microcircuit, in particular, the existence of a layers IV to VI to IV feedback loop [44], [46], [50]. Furthermore, a different degree of spatial modulation for inter-layer excitation and inhibition mimic the on-center off-surround arrangement of layers VI to IV projections [56]. In the simulations described below we assume that the LGN input to the lower layer is weaker (by a factor of 2) than the input to upper layer to account for the fact that thalamo-cortical synapses reaching layer VI are smaller in number than those reaching layer IV [45]. We also assume that latencies for inter-layer connections are longer than for intra-layer connections, thus accounting for the multisynaptic nature of this coupling. Our assumptions on the connectivity, external inputs and latencies are further commented upon in the *Discussion* section.

In order to analyze the role of the interlayer interactions in shaping the spatiotemporal dynamics of our model hypercolumn, we introduce a parameter 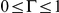 which homogeneously rescales the strength of excitatory and inhibitory connections between layers. For 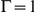 the interactions between the layers assume their maximum strength. For 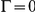 the layers are completely independent. In the following, we consider first the dynamics of the network at full coupling strength, 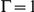.

### Orientation tuning and contrast dependence of induced response

In absence of “visual” stimuli (contrast level 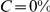), the model hypercolumn is driven only by the non-selective background input. The resulting spontaneous activity is heterogeneous across the neurons with average firing rates of 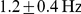 and 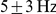 for excitatory and inhibitory neurons, respectively. Differences in the spontaneous firing rate distributions for upper and lower layers are not statistically significant at the 5% confidence level. The spontaneous firing of the neurons is highly irregular due to the stochasticity of the inputs. For instance, the average coefficient of variation (CV) of the interspike histogram of excitatory and inhibitory neurons in the upper layer is 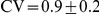. More details about rate and CV distributions can be found in Figure S1.

The profile of the activity induced by an oriented stimulus in both layers, is localized and centered at an angular coordinate corresponding to the stimulus orientation. Hence, the neuronal responses are selective to the stimulus orientation. The tuning curves of individual neurons display some heterogeneity in their broadness, as exhibited by distributions of peak response rates, circular variance and skewness of the tuning curves (reported in Figure S2).

**Figure 2:**
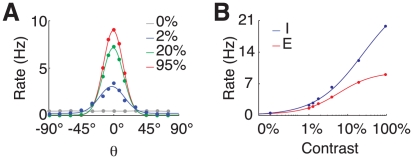
Response tuning and contrast response. A: tuning curves for different contrast levels (re-centered average over *N_E_* = 4000 excitatory neurons in upper layer). Solid lines represent Gaussian fits. B: contrast response functions. Blue curve: average over *N_I_* = 1000 inhibitory neurons in the upper layer. Red curve: average over *N_E_* = 4000 excitatory neurons in the upper layer. Solid lines represent hyperbolic ratio fits.

Figure 2A displays the population average tuning curve for various contrast levels for excitatory neurons in the upper layer. Comparison between tuning curves at different contrasts reveals that tuning width is approximately contrast invariant and that the larger deviations are observed for small contrast levels (tuning curves normalized to the peak are plotted in Figure S3). This invariance is achieved as an effect of noise in synaptic inputs [57], [58].

The preferred responses of the excitatory neurons vary non-linearly with the contrast as depicted in Figure 2B, where the population average Contrast Response Functions (CRFs) are plotted for excitatory neurons in the upper layer. It can be fitted by an hyperbolic ratio function (see *Methods* section), with mid-range contrast 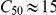
% and an exponent of 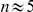 (upper layer neurons). This nonlinear dependence stems from the fact that increased sensory-driving yields larger inhibitory neurons activity which in turn is responsible for the saturation of the excitatory population response [59]. The CRFs of inhibitory neurons show a much weaker tendency to saturation at large stimulus contrasts which is due to the logarithmic dependency on the contrast of their external input. The CRFs of single neurons are heterogeneous, in qualitative agreement with experimental reports [60] (see Figure S4). The contrast response functions of the lower layer are homologous, but the induced responses are approximately twofold smaller, due to the weaker LGN driving.

### The dynamical state of the network depends on the stimulus contrast

For zero contrast, the synchrony level in the spontaneous neuronal activity is small, as denoted by a small value of the synchrony factor 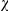. This factor, defined in the Methods section, quantifies global synchrony over a network and is bounded between 0 and 1. For a network of size 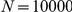, the synchrony factor for spontaneous activity assumes the value 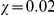. Furthermore, it vanishes consistently as 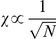 for larger network sizes, allowing us to classify formally the state of the network as “asynchronous” (see later discussion, Methods section and [34], [35]).

**Figure 3:**
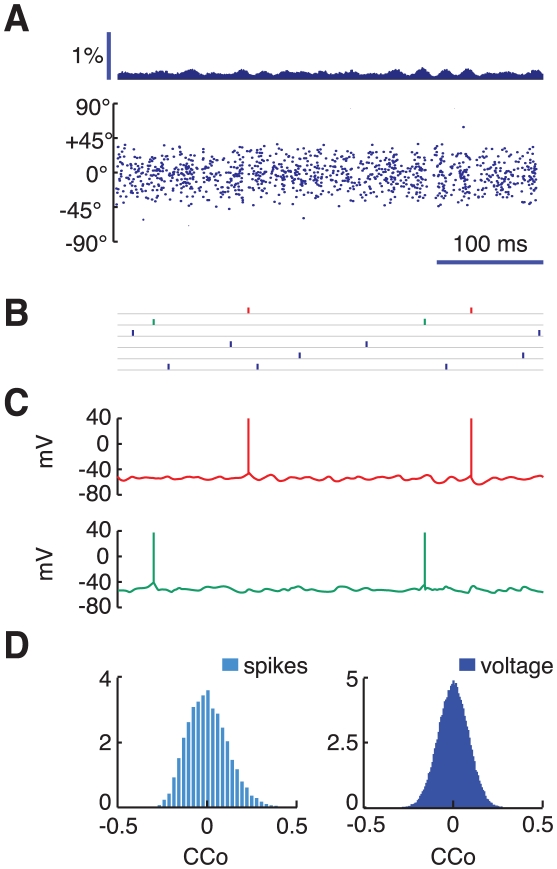
Low contrast dynamics. Dynamics of the upper layer for the presentation of a 2%-contrast stimulus. A: raster plot of the excitatory population activity and associated time-histogram of the rate of spiking cells. The histogram bar heights denote the fraction of upper layer excitatory cells that fire in the bin. Bin-size is 2 ms. B: spike trains of 6 excitatory cells highly activated by the presented stimulus. C: membrane potential traces for two neurons stimulated simultaneously at close-to-preferred orientation (2 top neurons of Panel B in red and green). D: pairwise correlations between spike trains (left, cyan histogram) and membrane potentials (right, blue histogram) of highly active neurons.

**Figure 4:**
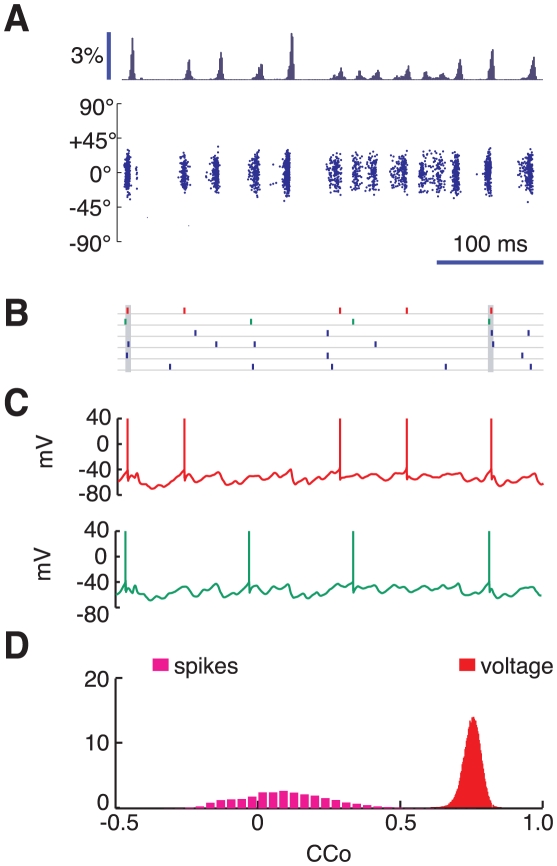
High contrast dynamics. Dynamics of the upper layer for the presentation of a 95%-contrast stimulus. A: raster plot of the excitatory population activity and associated time-histogram of the rate of spiking cells. The histogram bar heights denote the fraction of upper layer excitatory cells that fire in the bin. Bin-size is 2 ms. B: spike trains of 6 excitatory cells highly activated by the presented stimulus. C: membrane potential traces for two neurons stimulated simultaneously at close-to-preferred orientation (2 top neurons of Panel B in red and green). D: pairwise correlations between spike trains (magenta histogram) and membrane potentials (red histogram) of highly active neurons.

**Figure 5:**
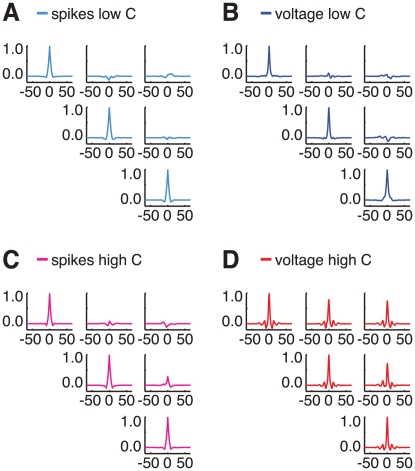
Pairwise crosscorrelations of spike trains and membrane potentials. Autocorrelograms and pairwise crosscorrelograms of spiking activity and membrane potentials for three upper layer excitatory neurons. A: spiking activity, low contrast, C = 2%. B: membrane potential, low contrast, C = 2%. C: spiking activity, high contrast, C = 95%. D: membrane potential, high contrast, C = 95%. Auto- and crosscorrelograms are normalized (for zero time-lag, autocorrelograms peak at one and crosscorrelograms at the correlation coefficient). The units for the time-lag axis are ms. Colors are as in Figures 3D and 4D. Rows and columns correspond to different neurons. The angular coordinates of the three neurons are 0°, −10° and 10°.

The single neuron and population responses of the network induced by visual stimulation are illustrated in Figure 3, for a low contrast stimulus (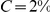), and in Figure 4, for a large contrast stimulus (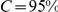). We focus first on the low contrast case. The raster plot of the spike activity of all the excitatory neurons in the upper layer is plotted in Figure 3A. It suggests that the firing is highly irregular (the mean CV of the upper layer excitatory neurons is 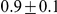, see Figure S1) and that the network activity of the network is only weakly synchronized. This is confirmed in Figure 3B where the spike trains of six upper layer cells stimulated within 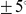 from their preferred orientation are plotted. The neurons fire without any noticeable synchrony. Figure 3C displays the voltage traces of two of these neurons. The comparison between the sub-threshold fluctuations in the two traces does not reveal any significant correlation. To further quantify the correlations in the supra and subthreshold activity of the neurons we compute the zero delay pairwise correlation coefficients (CCos) of the spikes and the membrane potential traces for a large number of pairs formed by highly active neurons with preferred orientation within 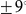 from the presented stimulus (see *Methods* section and Figure S5 for details). The resulting histograms are shown in Figure 3D (spikes: left, cyan color; voltage: right, blue color). They are peaked around zero with a mean statistically indistinguishable from zero (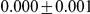 for spikes and voltage). Almost all the CCos are weak for the spikes as well as for the voltage traces (CCos larger than 0.25 occur only for 2% of the pairs when considering spike CCos, and for 0.1% of the pairs when considering voltage trace CCos). These results are consistent with a very weak synchronization in the network activity. This is in line with the small value of the synchronization factor, which is only 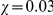. Auto- and crosscorrelograms of spike trains and membrane potential traces of three representative neurons are also shown in Figure 5A,B. The pairwise crosscorrelograms of both spikes and voltages do not display any persistent oscillatory component, even when two cells share a same orientation preference.

The dynamical state of the network is qualitatively different for a high contrast stimulus. For 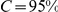 the neurons are engaged into a collective pattern of synchronous oscillations in contrast to what happens for 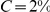. This is clear from the raster plot in Figure 4A. Figure 4C plots the membrane potential traces of two neurons. Comparison of these traces suggests that now the subthreshold membrane fluctuations of the neurons are strongly correlated across the network. As a matter of fact, the synchrony factor, 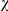, which characterizes the degree of synchrony in the subthreshold activity at the network level, is 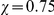. However action potentials are much less synchronized, as suggested by the comparison of the spike trains of the six neurons plotted in Figure 4B: although multi-neuron coincidences in firing (denoted by vertical grey bars) can be detected, the overall synchrony is weak. This substantial difference in the strength of the pair correlations in supra and subthreshold activities is clear in Figure 4D. All the CCos of the subthreshold membrane potentials (red histogram) are large and sharply distributed around 0.75 (standard deviation of 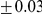) whereas the distribution of the spike trains CCos (magenta histogram) has a mean which is only 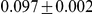. Remarkably, the firing activity continues to be highly irregular, despite the high degree of synchrony (mean CV of upper layer excitatory neurons is 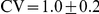, see Figure S1). Auto- and crosscorrelograms of spike trains and membrane potential traces of three representative neurons are shown in Figure 5C,D. The pairwise crosscorrelograms of *voltages* display now a clear oscillatory structure, which is however completely damped after only two or three cycles. Note that oscillatory correlations are evident even when the difference of preferred orientation is large (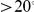). Note however that pairwise crosscorrelograms of *spike trains* do not display any marked oscillation even when the two considered cells have similar preferred orientations. We stress that the small mean value CCos and the lack of a clear oscillatory structure in the crosscorrelograms for spike trains, in both the low and the strong contrast case, is associated to the irregularity and the sparseness of single neuron firing.

**Figure 6:**
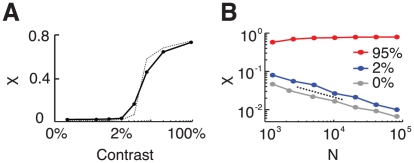
The Measure of synchrony as a function of the contrast and different network size. A: The synchrony measure, 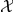, increases abruptly with the stimulus contrast *N* = 10000 (solid line) and *N* = 40000 (dotted line). B: The synchrony measure 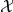 as a function of the network size for spontaneous activity (zero contrast, grey line), low contrast (blue line) and high contrast (red line). The dashed line corresponds to a power-law decay with exponent −0.5, denoting a regime of asynchronous activity.

These results indicate that synchrony in the population activity increases with the contrast. As a matter of fact, the synchrony measure 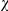 varies abruptly around a contrast value of 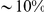, as shown in Figure 6A. This is even sharper with larger network sizes (compare in Figure 6A, the solid line which is for 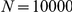 with the dashed line which is for 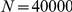). Moreover, a systematic analysis of the dependency of 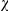 on the size 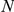 reveals that for 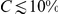, 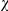 (*low contrast regime*) vanishes consistently with 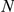, 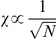, while for 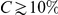 (*large contrast regime*) it converges toward a constant non zero value (Figure 6B). Hence, the network operates in qualitatively different regimes at low and high contrast. Whereas the network state can be classified as asynchronous in the low contrast regime (and in the spontaneous activity regime), it is synchronous in the high contrast regime. This sharp variation of synchrony is indicative of a phase-transition occurring for increasing contrast, due to an increased drive to the network (see Discussion).

**Figure 7:**
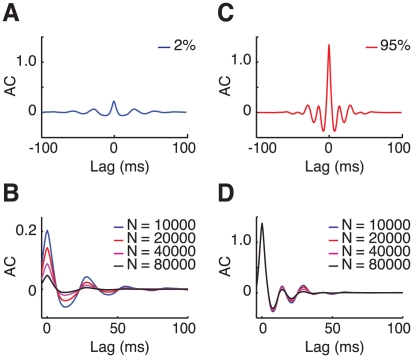
The autocorrelograms of the local field potentials. A–B: low contrast, *C* = 2%. C–D: high contrast, *C* = 95%. Scalings of non-normalized autocorrelograms are shown in B and D. In both cases the damping of secondary peaks is faster for larger network sizes. Zero-lag autocorrelation vanishes for large sizes at low contrast but not at high contrasts. Non-normalized autocorrelations are measured in *nA* ^2^.

To characterize further how the population dynamics depend on the contrast we compute the autocorrelation, 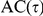 of the LFP signals induced by stimuli oriented at the preferred orientation of the recording site (see *Methods* for the way we define the “LFP” signals in the framework of our model and Figure S6 for examples of LFP traces). The result for low contrast, 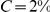, is plotted in Figure 7A, B. The amplitude of the (non-normalized) AC at zero delay, 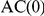, is small and decreases with the network size as 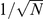. Similarly, the small oscillatory component of the AC disappears gradually for increasing network sizes (Figure 7B). This is because the network state is asynchronous and in a larger network more cells contribute to the LFP signal (see also *Methods* section).

The fact that at high contrast, 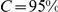, the network is engaged in collective synchronous activity is manifest in Figure 7C,D: 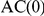 is now large, it does not vanish in the large 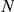 limit and is almost independent of 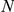 for 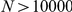. However, and remarkably, the induced dynamics exhibit a spatio-temporal structure which is more complex than a periodic regular oscillation of the population activity: the time interval between consecutive episodes of synchronous activity displays cycle-to-cycle fluctuations as can be observed in the raster plotted in Figure 4A). As a result, the LFP autocorrelogram is rapidly damped. Although it displays some secondary peaks their amplitudes are very small as shown in Figure 7C. The damping of the AC oscillations is even faster for larger network sizes (Figure 7D). Note that autocorrelations for intermediate contrast values are also rapidly damped (see Figure S6). A moderate tendency to period doubling, manifested by a second autocorrelogram peak slightly larger than the first autocorrelogram peak, is observed in our model. To our knowledge this has not been observed in experimental studies. However, this feature disappears for larger network sizes or stronger inter-layer coupling.

LFPs induced by non-preferred stimulus directions display as well oscillatory components, for both low and high contrasts. Induced LFPs are correlated over the entire ring network as revealed by crosscorrelation analysis, confirming that sub-threshold coherence can exist independently from correlations in spiking activity (see Figure S6).

**Figure 8:**
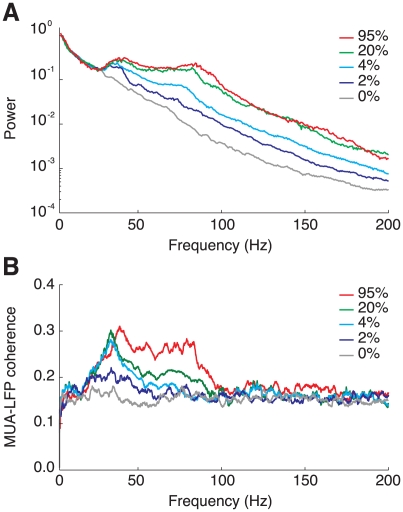
Spectral properties of the LFP and MUA for different contrasts. A: Power spectra for the LFP induced by a stimulus at preferred orientation. Isolated peaks do not appear even for very high contrast stimuli. B: Average coherence spectra between the MUA and the LFP induced at a same location by a stimulus at preferred orientation. MUA-LFP coherence and LFP power are modulated by contrast changes in the same broad frequency range in the gamma band (30–100 Hz).

Finally, we consider the spectral properties of induced LFPs, and their relation with MUA observed at a same location. The dependency on the contrast of the power spectra of the LFPs induced by preferred-orientation stimuli is shown in Figure 8A. The low-frequency part of the power spectra is weakly dependent on the stimulus contrast. Rather, it is shaped by the properties of cortical background activity, modeled as a stochastic Ornstein-Uhlenbeck noise with a frequency cutoff (see *Methods* section and [61]). This should be compared to the boosting of the power as the contrast increases for frequencies 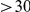 Hz. Although the network activity becomes much more synchronous at large contrast as explained above, power spectrum modulations are not limited to narrow peaks, but, even at the highest contrast, the whole frequency range comprised between 30 and 100 Hz is boosted. In this same broad frequency range in which contrast-dependent power modulations occur, the LFP displays phase-synchronization with the MUA at a same location, as measured by a MUA-LFP coherence increasing with contrast (see Figure 8B). Interestingly, the MUA-LFP coherence, even at full contrast, rises only at an average peak level of approximately 0.3, compatible with physiologic ranges of synchronization [9], [62]. This can be explained by the random-like variability of single neuron firing –inherited by the MUA signal, which reflects the spiking activity of only a limited number of single units (see *Methods* section)–, but also by the lack of phase autocoherence in the LFP signal itself (see [63]).

The spatio-temporal structures of the induced activity in the lower and in the upper layers are similar. In our simulations, the lower layer average firing rate is approximately half of that in the upper layer, reflecting weaker driving from LGN. Cross-correlation analysis of the LFPs in the two layers shows that the lower layer oscillations lag behind those in the upper layers (see Figure S7). Note that larger response latencies in deep layers have been experimentally observed in specific conditions [64], [65]. However, the multi-layer structure in our model is too schematic to capture quantitatively such inter-layer relations. In particular, the difference in response rate and the exact locking pattern between layers depend in our model on the parameters of LGN input and inter-layer coupling. On the contrary, the synchronization and the fast decorrelation of induced oscillations are robust against changes in these parameters (see later Discussion).

### The role of inter-layer coupling in destroying the temporal coherence of the oscillations

In order to explore the role played by the inter-layer interactions, we investigate in the following how the dynamics in the high contrast regime is affected by a change of this coupling. More specifically, we rescale the peak conductances of all the synapses between cells in different layers by a same factor 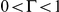 (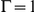 and 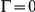 correspond respectively to fully coupled and fully decoupled layers).

**Figure 9:**
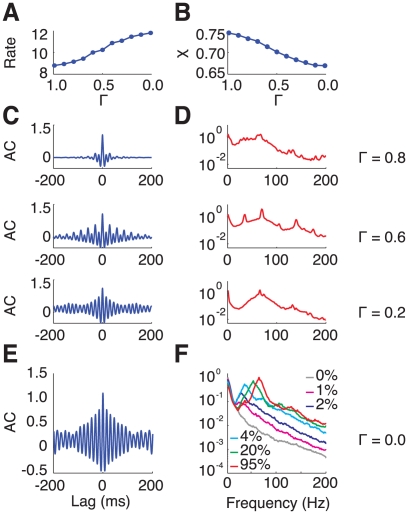
Effects of the layer decoupling on the dynamics of the hypercolumn. Changes for decreasing inter-layer coupling and for a stimulus at high contrast with preferred orientation. A: population average peak firing rate for the excitatory neurons in the upper layer. B: synchrony level 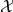. C: autocorrelograms of LFPs for intermediate strengths of the inter-layer coupling (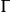 = 0.8, 0.6 and 0.2). D: corresponding LFP power spectra. E: autocorrelograms of LFP for preferred stimulation at high contrast for the case of fully uncoupled layers (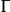 = 0∶0). F: corresponding LFP power spectrum. Spectra are also plotted for lower levels of contrast and are characterized by a narrow peak at a contrast-dependent frequency.

Upon layer-decoupling the mean firing rate of the excitatory and inhibitory cells increases in the upper layer (Figure 9A). However response rate changes are highly heterogeneous across cells and, in some cases, the peak rate is even slightly reduced. An analogous heterogeneity is observed in the changes in the preferred orientation, skewness and tuning width. However, even though changes after complete layer decoupling can be significant for specific cells, the distribution of tuning curve parameters over the entire upper layer excitatory neurons population is only weakly altered. Details are shown in Figure S8.

Another effect of layer decoupling, albeit moderate, is that the degree of synchrony in induced activity decreases monotonically with 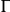 (Figure 9B). For instance, the synchrony factor is 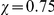 for 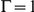, but decreases to 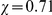 when 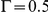, and drops further to 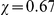 for fully decoupled layers.

The most striking consequence of the reduction in inter-layer coupling is the progressive qualitative change in the shape of the LFP autocorrelograms and power spectra as 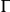 decreases. This is depicted in Figure 9. For 80% coupling strength (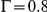), the autocorrelogram of LFP and the corresponding power spectrum are similar to what is found in the fully-coupled case (fast temporal decorrelation and broad plateau-like peak in the gamma spectral band, see Figure 9C, D). However, for a 60% coupling strength (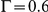), the LFP temporal decorrelation becomes considerably slower and the envelope of the autocorrelogram displays amplitude modulations indicating that the LFP signal is quasi-periodic. In parallel, the gamma-band spectral plateau is replaced by a system of narrow peaks at incommensurate frequencies. The raster plot of activity (not shown) continues to display a temporally irregular oscillation; however spatial fluctuations in the width of consecutive bumps of spiking activity are reduced with respect to the fully-coupled case. For further reduction of the interlayer coupling to 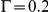, the LFP autocorrelogram starts revealing periodicity of the signal over long time scales. The multiple narrow spectral resonances merge into a single prominent resonance in the gamma-band and secondary harmonic peaks also appear. Finally, for 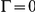 (Figure 9E, F), the LFPs are still substantially autocorrelated after several hundredths of ms. Spectra in the synchronous regime are harmonic at any contrast level. More details about the high contrast regime for completely uncoupled layers are presented in Figure S9.

Interestingly, qualitative modifications of the population dynamics when 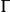 is varied do not occur in the low contrast regime, in which collective oscillations do not develop. As a matter of fact, independently of the coupling strength 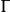, induced activity is asynchronous. Spiking and LFP responses to a low contrast stimulus between completely uncoupled or fully coupled layers are practically indistinguishable (not shown).

### Stimulus repetition and chaotic sensitivity to initial conditions

Up to now we have focused on the response of the network to a time independent stimulus. Here we show that the inter-layer coupling also strongly affects the response of the model hypercolumn induced by an external input which varies periodically, representing visual stimuli to V1 in the form of flashed or drifting gratings. In this situation, we characterize the neuronal responses by means of peristimulus time histograms (PSTHs) which express the probability of observing the firing of a spike at a given time relative to the onset of each stimulus presentation (see *Methods* section). In the following, we focus on high contrast stimuli.

**Figure 10:**
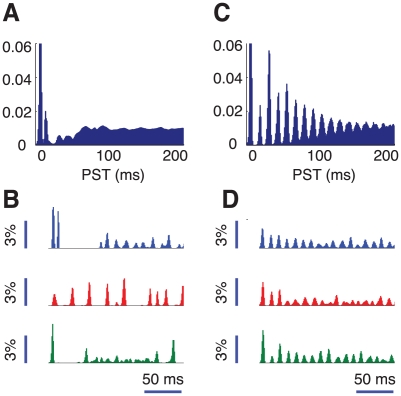
Short-term response. Population firing responses to repeated presentations of a high contrast stimulus for fully coupled layers (A–B, 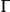 = 1) and for fully uncoupled layers (C–D, 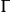 = 0). A and C: peristimulus-time (PST) histograms, based on the firing responses of 500 cells to 1000 presentations of stimuli with optimal (or close to optimal) orientation. B and D: examples of upper layer excitatory population responses for three presentations of the same stimulus.

The PSTH for 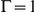 is shown in Figure 10A. At the onset of the stimulus the probability of firing increases sharply, followed by a transient phase of reduced firing. This feature is not evident in experimental PSTHs. It is due to the strongly synchronous recruitment of recurrent inhibition which follows the initial burst of activity, triggered by the rise of external inputs (instantaneous in our model). Notwithstanding, after a few tenths of a ms the firing probability rises again and remains then almost constant. This reflects the fact that the population responses are highly variable across trials as is clear in Figure 10B. In each trial the response of the network consists of a sequence of episodes in which the neurons tend to fire together. However, there are substantial trial-to-trial fluctuations in the timing of these episodes and their amplitude (i.e. the numbers of recruited cells). Consequently, although the presentations of the stimulus do give rise to synchronous activity, the PSTH histogram averaged over many trials is almost flat after a peri-stimulus time on the order of the short temporal decorrelation time of the induced oscillation.

In contrast, for fully decoupled layers (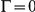), the PSTH averaged over many trials exhibits a long-lasting, although damped population oscillation, as plotted in Figure 10C. This is because when the layers are decoupled the oscillations generated inside the layers are close to being periodic and they maintain coherence over several hundred milliseconds. Hence the timing of the oscillations does not fluctuate much across trials (Figure 10D). Population oscillations are thus masked by averaging across multiple stimulus repetitions only after many cycles.

**Figure 11:**
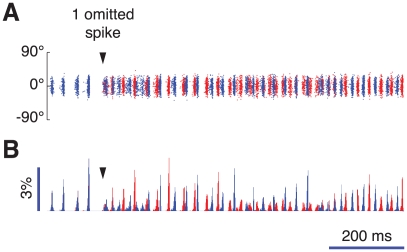
Chaotic sensitivity to a single spike perturbation. A black triangle denotes the time of a small perturbation to the network dynamics (for 95% of contrast stimulus and fully-coupled layers, 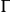 = 1), in which a single spiking event is omitted. Already after the second oscillation cycle, the unperturbed and perturbed population dynamics have diverged, as visualized by the raster plot (A) and the population rate histogram (B) of the upper layer excitatory population. Blue color denotes unperturbed dynamics and red color perturbed dynamics.

The large trial-to-trial variability displayed by the network for 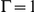 (Figure 10C) indicates a strong sensitivity to initial conditions (i.e. the network configuration at the onset of the stimulus). To further illustrate this sensitivity, we perturb the dynamics of the system by omitting artificially a single spike in a single neuron (out of 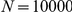) at the center of the bump of induced activity and we compare then the perturbed and the unperturbed dynamics. The results of this numerical simulation are illustrated by Figure 11. As visible from the raster plot (Figure 11A) and the population rate histogram (Figure 11B) of the upper layer induced activity (at full contrast), the perturbed and the unperturbed collective oscillations can be distinguished already after one oscillation cycle. After a few cycles, they have completely diverged. Such extreme sensitivity to perturbations or initial conditions is strongly indicative of dynamical chaos [66]. The sequence of states observed in our model for decreasing 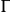 (from irregular to quasi-periodic to periodic, see Figure 9C,D) also suggests that chaos might emerge for strong inter-layer coupling and that its onset might occur according to a quasi-periodic scenario [66], [67]. This is indeed one of the possible scenarios for the transition to chaos occurring in a related rate model [41]. As we discuss in detail in the Text S1 and in the Figure S10, the chaotic nature of the dynamics of the network for 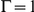 and high contrast stimuli can be assessed by an estimation of its largest Lyapunov exponent 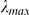 [66]. A positive value of this Lyapunov exponent is the manifestation of deterministic chaos, denoting exponentially fast separation of trajectories. Using techniques of non-linear time-series analysis [68] applied to very long stationary time-series of LFP from our model (see *Methods* section, Text S1), we obtain the estimate 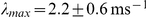, which is indeed positive. Interestingly, the dynamics of the network with uncoupled layers (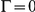) fails to display a positive Lyapunov exponent (see Figure S10), and it is therefore non chaotic, confirming the role of inter-layer coupling in inducing (see also the *Discussion* section).

## Discussion

### The structure of the model

#### Multi-layer architecture

The reduction of the full multi-layer structure of primary visual cortex (a cartoon of which is shown in Figure 1A) to a simpler two-layer network (Figure 1B) is a drastic simplification. Throughout this paper, we have emphasized that the two main cortical thalamo-recipient layers, i.e. IV and VI [42], [43], [45] are included within distinct model layers, corresponding respectively to the upper and the lower ring in our network architecture. We do not include separate rings for each of the six cortical layers. However, in order to reflect the poly-synaptic nature of the pathway from cortical layer IV to VI –passing through layers II/III and V [45], [46], [49]–[51]– we have made the latency of the connections from the upper to the lower model layer larger than for the connections from the lower to the upper model layer. The incorporation of additional layers within our model is in principle possible, but at the price of increasing further an already large number of parameters. Our choice of introducing just two layers was guided by the need to keep the model as simple as possible, while retaining a multi-layer structure.

In the simulations described above, the external drive is smaller to the lower layer than to the upper layer. This choice was motivated by the fact that thalamic projections toward layer IV are more numerous than toward layer VI [45]. Nevertheless, it should be noted that layer VI neurons have dendritic arborizations extending into layer IV where they can receive additional thalamo-cortical inputs [69]. However, as illustrated in Figure S11, the behavior of the network remains qualitatively the same, if one adopts identical external drives for the two layers. A second aspect that we have neglected about differences in the external drive to different layers, is the fact that the size of receptive fields depends on laminar location. In particular the receptive fields of layer VI neurons can be larger than the ones of layer IV neurons [70], [71]. However, a proper description of the stimulus-size dependence of the inputs would require as well to take into account horizontal interactions between different layer IV receptive fields fitting into a same larger layer VI receptive field, a modeling aspect that we hope to address in future investigations.

#### Connectivity

In our model intra-layer excitation is modulated more strongly with angular distance than intra-layer inhibition. However, the probability of inhibitory connections is larger than the probability of excitatory connection at any angular distance (Figure 1E). In addition, we choose conductance parameters such that individual inhibitory PSPs are stronger than excitatory PSPs [72]. Thus, intra-layer inhibition dominates intra-layer excitation at any distance. As a consequence, in the regimes explored in this paper, recurrent interactions are not sufficient to generate a tuned response by themselves. However they sharpen the tuning already present in the spatially-patterned feed-forward LGN input. We use probabilities of connection compatible with the wide ranges reported by [73], [74]. Other studies, like [72], find a larger probability of inhibitory connection. We verified however that the qualitative properties of the induced regimes of activity are preserved when inhibitory connections are consistently densified (see Figure S12).

The dominantly inhibitory nature of mutual local interactions is essential in our model for the emergence of prominent collective oscillatory behaviors in our network. Oscillations are generated by mutual delayed interactions between inhibitory neurons, according to a standard mechanism already described in [25], [27]–[30]. In our model, excitatory neurons are not required for the generation of oscillations. Excitatory neurons are entrained by the oscillation paced by inhibitory cells. Indeed, if the activity of excitatory neurons is completely suppressed, or if synapses from excitatory to inhibitory neurons are removed, while increasing the drive to inhibitory neurons in order to maintain their rate of activity unchanged, the oscillations continue to exist and their frequency increases of less than five percent (see Figure S13). We mention here that an alternative scenario exists in which the inhibitory-to-excitatory-to-inhibitory neurons feedback loop plays an active role in the generation of synchronous oscillations [27], [30], [75]–[77]. In this scenario delayed inhibitory feedback is still the cause of the oscillation, but the delay arise from the disynaptic nature of effective mutually inhibitory interactions, leading to a slower collective frequency. However, the analysis conducted in Figure S13 clarifies that the scenario implemented in our model relies primarily on inhibitory interneurons alone.

Inter-layer connections in our model are as dense as intra-layer connections, but inter-layer excitation is more sharply modulated than intra-layer excitation. This results in a smooth arrangement of vertical excitatory synapses reminiscent of the organization of cortex into a continuum of anatomical columns without rigid boundaries [78]. This arrangement is critical for the fast temporal decorrelation of induced oscillations at high contrast (see below).

Whereas the net inter-layer coupling is moderately excitatory in a local center, it is inhibitory in the surround, as a combined effect of the broad profile of inter-layer inhibition and of the fact that lower-to-upper excitation toward inhibitory neurons (i.e. disynaptic inhibition) is less sharply modulated than lower-to-upper layer excitation toward excitatory neurons. This is required in our model to account for the increase in mean firing rate observed in layer inactivation experiments [79] (case 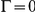 in our model).

### The low and high contrast regimes

Most of the simulations described above were performed in networks with a significantly smaller number of neurons (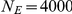 excitatory neurons and 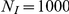 inhibitory neurons per layer) than in a real hypercolumn in V1. However, we checked that our results are robust against increases in network size. In particular, this is the case for the existence of two dynamical regimes induced respectively by low and high contrast stimulations and for the two distinct mechanisms underlying the fast temporal decorrelation and broad-band spectral modulations in these two regimes.

In the low contrast regime, the dynamics are asynchronous. However, the network tends to resonate at a specific frequency, producing an increase of power in the gamma frequency band, without developing stable oscillations. Weakly coherent oscillatory modes are excited only transiently by local noise and then quickly damped.

On the other hand, in the high contrast regime the network activity is synchronous. However the collective rhythm undergoes random variations in the time interval between consecutive activity episodes in the network. This temporal irregularity is not due to local noise (note that, in our model, recurrent inputs dominate over feed-forward inputs at low as well as at full contrast). It is produced intrinsically by the dynamics by virtue of the interaction between distinct oscillating populations localized in the two subnetworks representing different depths in the cortical section. This results in rapid temporal decorrelation of the induced activity.

The contrast at which the transition between these two regimes takes place depends on the strength of fluctuations in the background noise. For our choice of parameters, the transition occurs for 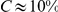. However, as discussed in detail in Figures S14, S15 and S16, if the variance in the LGN input current is increased consistently without changing its mean value (Figure S14, parameters in Table S1), the transition can occur for an external drive, which is so large that it cannot be reached even for stimuli at full contrast (Figure S15). In such a condition, the induced activity is still asynchronous at high contrast and only transient oscillations can be detected (Figure S16), as in the recent modeling study by Mazzoni et al. [37].

It has been observed experimentally that the gamma-band synchronization of membrane potential fluctuations of nearby cells in V1 is larger in visually-induced activity than in spontaneous activity. Furthermore it is sustained over long stimulation durations, independently from stimulus properties or from the simultaneous observation of synchronized spiking activity. This leads to voltage crosscorrelograms with a manifest oscillatory component at gamma-range frequencies, damped quickly within only two or three oscillation cycles [80]. These observations are compatible with the occurrence of a transition between an asynchronous low contrast regime and a synchronous high contrast regime. Indeed, pairwise CCos between membrane potentials are small in the low contrast regime (Figures 3D and 5B), but large in the high contrast regime (Figure 4D and 5D), even if spike CCos are always small, in agreement with many experimental reports [5], [13], [81]–[86]. We remark that if the dynamics at high contrast would be asynchronous as the dynamics in absence of stimuli or for low contrast stimuli, then the pairwise crosscorrelations of *both* spikes and voltages should be weak. Therefore, the coexistence of weak correlations between spikes with stronger correlations between membrane potentials (displaying furthermore a damping oscillatory component) is suggestive of the existence of a synchronous, rather than of an asynchronous, regime. The dynamics at high contrast of our model, characterized by irregular spiking (leading to weak spike crosscorrelations) and by temporally irregular collective oscillations (leading to quickly damped oscillatory voltage crossocorrelograms) is therefore compatible qualitatively with the experimental regime observed in [80]. Conversely, this compatibility could not be claimed for the other two types of induced dynamics that our model can generate at full contrast, i.e. asynchronous, in the case of a large variance noise, or synchronous but approximately periodic (and therefore too slowly decorrelating), in the case of suppressed inter-layer interactions (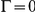).

### Synchronous chaos underlies the temporal decorrelation of the network collective oscillations in the high contrast regime

The rapid loss of temporal coherence of the synchronous induced activity at high contrast is a remarkable property of our model. Features of the model such as inter-layer inhibition, asymmetric interaction latencies in the lower-to-upper or in the upper-to-lower direction or different LGN driving levels to the different layers are not required for this decoherence to occur. In contrast, the strong local inhibition responsible for the local generation of the rhythm within each layer and the net excitatory interactions between neurons in close vertical alignment are crucial for this to occur. In fact, if the inter-layer excitation profile is altered by suppressing its modulation with orientation distance while keeping its average strength constant, the decorrelation does not take place (see Figure S17).

A similar mechanism underlies the temporal decorrelation of synchronous oscillations in the network models studied by [39]–[41]. These papers showed that collective oscillations induced in two populations of neurons by local delayed inhibitory feedback can lose coherence when the two populations interact in an excitatory manner. In [41], we studied a rate model consisting of two networks, each composed of one excitatory and one inhibitory populations. Each of the networks was able to sustain synchronous oscillatory activity by virtue of the local inhibition. We computed the maximum Lyapunov exponent of the system (see e.g. [66]) to show that it undergoes a transition to a chaotic dynamical state when the two networks are coupled by sufficiently strong excitatory connections. In this state the network displays synchronous activity, but instead of being periodic, the temporal variations of the network activity are chaotic and thus the oscillations that the network tends to develop lose temporal coherence within a few cycles. A network operating in such a regime is said to be in a *synchronous chaotic* state. In [39], [40] a single ring network with strong local inhibition was considered. The decoherence of the oscillations occurred as the network underwent a spontaneous clustering into groups of oscillating neurons effectively interacting in an excitatory manner.

In agreement with the positivity of its largest Lyapunov exponent, also the dynamics of our hypercolumn model in the high contrast regime displays typical features of chaos: exponentially fast damping of the local oscillations autocorrelograms (Figure 7C,D), spreading of the oscillation-related power over an extended continuous interval (Figure 8), and extreme sensitivity to initial conditions (Figure 10B and Figure 11). Therefore the decoherence of the population activity which occurs at high contrast stems in the present model from the fact that the network operates in a synchronous chaotic regime. We cannot exclude, obviously, that other mechanisms are contributing to the decorrelation of synchronous cortical oscillations. However, such a global decorrelation, characterized by the coexistence of elevated instantaneous synchrony and fast loss of collective phase autocoherence, could not be induced by local external noisy inputs, unless they are spatially correlated over a range matching the size of the local circuit which generates the ongoing soscillation.

We also conjecture that the underlying mechanism of synchronous chaos is very general as it occurs in models in which neurons are described in term of rate, integrate-and-fire or conductance-based dynamics, with a simplified as well as more complex multi-layer network architecture. We also conjecture that a similar mechanism should act in even more realistic models, incorporating for instance a two-dimensional spatial structure, similarly to the one used in [87], [88], provided that local inhibition is strong enough to induce local oscillations and that excitation couples these local oscillators at a longer range.

### Comparison with previous works

Chaotic dynamics as well as stable chaotic-like dynamics can occur in asynchronous states of activity [89]–[97]. In this cases, the network dynamics explores a high-dimensional manifold in the phase-space, while, in our model, the irregular sparse firing of many neurons give rise to collective synchronous chaos (SC) with a lower dimensionality [98]–[100] (the fractal dimension of the chaotic attractor is likely to be smaller than five, as discussed in Figure S10).

SC has also been found in previous models of local circuits in V1 which consisted of only one single network with a ring architecture. The model studied by Hansel and Sompolinsky in [101] considered one neuronal population coupled with excitatory instantaneous synapses. It displayed a SC state in some appropriate range of parameters. However, in this model, SC was sensitive to the incorporation of synaptic time constants since it was destroyed with the introduction of synaptic time constants as small as 0.5 ms. The model by the same authors considered in [34] considered two populations of neurons, one excitatory and one inhibitory, coupled via synapses with realistic synaptic time constants. The dynamics of the neurons were based on a Hodgkin-Huxley type model with several cellular and synaptic conductances. The pattern of connectivity had a “Mexican hat” with local excitation and broad range inhibition. Numerical simulations of the model showed that in an appropriate parameter range, the network settled in a SC state, characterized by strong temporal variability of the neural activity which was correlated across the hypercolumn.

In both of these models, the SC state was characterized by strong neuronal pairwise spike correlations and wide variability in the firing of individual neurons which was induced by the chaotic nature of the population activity. This is essentially different from what happens in our two layers hypercolumn, in which, in the SC state at high contrast, the spike pairwise correlations are only slightly larger than in the low contrast asynchronous state, whereas the degree of irregularity in the spike trains are similarly large in both states (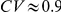). As a matter of fact, in the present model, the spike train irregularities are mostly due to the local noise generated by the external inputs and to a lesser extent by the internal dynamics. Voltage CCos are large due to macroscopically correlated chaotic sub-threshold fluctuations, but spike CCos are still small. Another essential difference is that in [34] the excitation was local and inhibition was broad, whereas the opposite is required in the present model, as well as in the single ring model in [40]. Last but not least, it is not clear to what extent the chaotic dynamics found in [34], [101] were specific to the model adopted there for the single neuron dynamics.

### Predictions and perspectives

The increase in synchrony of the activity with the contrast displayed by our model is in agreement with experimental results reported recently in monkey V1 [9], [18]. More generally we should expect that varying a feature of a stimulus in a way that increases the external drive on V1 network should have a similar effect. This is consistent with other recent results showing that varying the size of a visual stimulus [12] or attention [6], [11] strengthens the coherence in the activity of V1 neurons.

In the low and large contrast regimes identified in our model the increased gamma power in the LFP spectra is broadband. At low contrast, the loss of coherence of the oscillations in the LFP in a few tenths of a milliseconds is due to noise. At large contrast, it is a consequence of the chaoticity of the LFP time-series. The behavior of our model in both these regimes is compatible with recent results by Burns et al [63], because of its lack of sustained auto-coherence of induced oscillations.

Our simulations predict that infra-granular layer inactivation should globally affect the experimentally observed spectral properties of induced LFPs by enhancing its periodicity. Single-layer inactivation experiments based on pharmacological or local cooling techniques [79], [102], [103] or with optogenetic techniques [23], [24] might be used to test this prediction. Furthermore, manipulations in which the firing of a single additional spike is induced (or suppressed, analogously to the simulation of Figure 11) can be performed. Extreme sensitivity to single-spike perturbations was experimentally proved using such a manipulation in the case of asynchronous spontaneous cortical dynamics [104]. It would be interesting to repeat similar experiments in a stimulus-induced regime of oscillatory activity, in order to study the impact of the addition of a single spike on the time-course of ongoing LFPs.

In the present study we focused on the role of the interactions between cortical layers in promoting temporal decoherence of gamma oscillations via the generation of synchronous chaos in a network with the size of a typical classical receptive field in V1. It would be interesting to investigate whether horizontal interactions which extend at distances beyond the classical receptive field also contribute to the loss of temporal coherence via a similar mechanism when the visual stimuli are extended. The basic two-ring network developed in this paper can be replicated into a bi-dimensional architecture including long-range excitatory interactions in order to investigate this potential contribution. This framework can be also applied to assess how the phase relationship between activity at different locations in V1 (e.g. between center and surround of an extended stimulus) depend on the polarity of long range interactions. Furthermore, an additional source of decorrelation might be inter-areal interactions occurring at an even longer range.

Finally, we have here considered temporal decorrelation induced by excitatory interactions between populations oscillating due to delayed mutual inhibition between interneurons. It would be interesting to investigate whether a similar decorrelation phenomenon can arise when the mechanism for the local generation of oscillations is different, and is based for instance on circuit loops with active involvement of pyramidal cells [27], [30], [75]–[77], [105]–[107].

## Methods

Our model of a functional hypercolumn in V1 consists of two interacting rings of neurons, an upper and a lower ring, each comprising 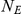 excitatory and 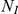 inhibitory neurons connected recurrently. We denote by 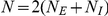 the total number of neurons in the network. Each neuron is labeled by its location on the ring to which it belongs; i.e. by an angular coordinate 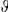, ranging conventionally from −90 to +90 degrees [53], [54]. All the neurons receive an external input composed of two contributions. One represents the LGN input to V1. It depends on two parameters 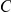 and 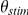 corresponding to the contrast and the orientation of a visual stimulus. The other contribution accounts for the background inputs V1 receives from subcortical regions.

### Single neuron dynamics

We use single-compartment Exponential Integrate-and-Fire model neurons (EIF; [108]). In this model the membrane potential 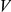 is given by the equation:
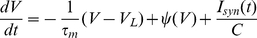
(1)where 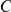 is the membrane capacitance, 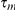 the membrane time-constant, 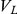 the leak potential, 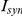 the total synaptic input current to the neuron. The function 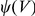 is:
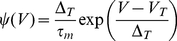
(2)For a constant input above a threshold current (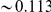 nA for the parameters adopted here) the solution of (1) diverges to infinity in finite time. This divergence is identified with the firing of a spike. The parameters 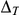 and 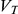 characterize how sharp the initiation of the spike is and the voltage at which it occurs. The spike downswing is not explicitly modeled. After each spike event, the voltage needs to be reset. A refractory period must then follow.

We model this refractoriness in a different way for excitatory and inhibitory neurons. In the case of excitatory neurons, following the emission of a spike at time 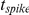, the parameters 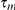, 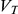 and 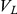 are updated according to the equations [109],
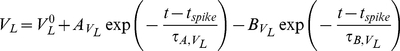
(3)

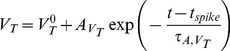
(4)

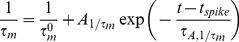
(5)The membrane potential is reset to a value 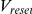 which is sub-threshold. Furthermore 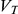 is strongly depolarized after a spike. Therefore the event that two spikes are closely emitted in time by a same neuron is extremely unlikely and, in practice, never occurs.

For inhibitory interneurons, we use a “hard” refractory period instead, suspending the numerical integration for a time 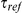 after voltage reset [108]. Therefore, 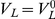, 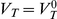 and 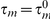.

Parameters for excitatory neurons are chosen to coincide with fits of pyramidal neurons traces, following [109]. We use analogous parameters for inhibitory neurons, apart from halved membrane capacitance and time constant 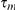, consistent with experimental evidence [110] and fits of interneuronal traces presented in [111]. All single neuron parameters are given in Tables 1 and 2.

**Table 1:** Parameters for model neurons.

	Excitatory	Inhibitory
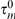	23.30 ms	11.65 ms
*C*	0.26 nF	0.13 nF
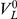	−57.8 mV	−57.8 mV
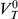	−45.2 mV	−45.2 mV
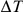	1.2 ms	1.2 ms

Parameters (without time dependency) of model excitatory and inhibitory EIF neurons.

**Table 2:** Soft refractoriness parameters.

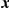	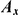	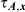	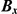	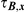
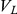	22.9 mV	14.7 ms	13.5 mV	76.2 ms
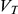	10.0 mV	17.7 ms	—	—
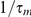	0.14 ms^−^1	14.3 ms	—	—

Parameters of time-dependent after-spike relaxation of excitatory EIF model neurons.

### The synapses

We use three kinds of synaptic currents, modeling inhibitory (GABA-type), fast excitatory (AMPA-type) and slow excitatory (NMDA-type) synaptic inputs. No voltage dependence is introduced for the parameters of the slow excitatory synaptic current. A spike in an inhibitory pre-synaptic neuron evokes a GABA-type post-synaptic potential (PSP) in all the post-synaptic neurons; a spike in an excitatory presynaptic neurons evokes composite AMPA- and NMDA-type PSPs.

The synaptic current produced by a single incoming spike is described as 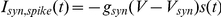, where 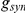 is the peak synaptic conductance, 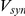 the reversal potential of the synapse (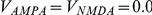 mV, 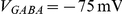). Denoting as 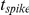 the time of pre-synaptic firing and with 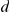 the synaptic latency, the function 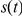 is:
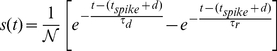
(6)where the constant 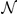 is such that it normalizes to unity the peak of 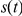. All the synaptic conductances in the network are calibrated to give unitary PSPs at resting potential in a range compatible with experimental observations [72].

The values of the synaptic times and synaptic peak conductances are given in Table 3, for a network including 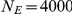 excitatory neurons and 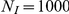 inhibitory neurons per layer. Synaptic peak conductances are rescaled for larger networks, according to Eqs. (8) and (9). Synaptic latencies are given in Table 4.

**Table 3:** Synaptic time-constants and efficacies.

	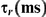	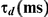	g (nS)	PSP (mV)
AMPA on excitatory	1	3	1.0	0.84
AMPA on inhibitory	1	3	1.5	2.07
GABA on excitatory	1	4	4.0	1.13
GABA on inhibitory	1	2	4.0	1.36
NMDA on excitatory	3	80	0.14	0.50

Synaptic parameters for a network of *N_E_* = 4000 neurons and *N_I_* = 1000 neurons: synaptic rise (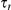) and decay (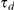) times, peak synaptic conductance (*g*) and peak postsynaptic potential *PSP*.

**Table 4:** Synaptic latencies.

	*d* (ms)
Intra-layer synapse	1.0
Inter-layer synapse (upper to lower layer)	3.0
Inter-layer synapse (lower to upper layer)	1.0

Synaptic latencies (*d*) depending on the relative position of pre- and post-synaptic neurons.

### Network connectivity

Each of the two layers of the hypercolumn is modeled by a ring-network [34], [53], [54], [112]. Unless specified otherwise, the simulations described in this paper were performed for a network comprising 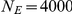 excitatory cells and 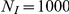 inhibitory cells per ring, for a total of 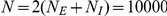 neurons in the hypercolumn. Note that a very similar network architecture was used in [113], [114] but with a completely different interpretation.

Intra-layer and inter-layer excitatory and inhibitory connections are random. The probability of connection between two neurons is spatially modulated and depends on the angular coordinates 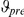 and 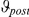 of the pre- and post-synaptic neurons. It also depends on the nature (excitatory or inhibitory) of pre- and post-synaptic cells and on their absolute (lower or upper layer) and relative (intra-layer or inter-layer) depth. All the profiles of connection probability are parameterized as:
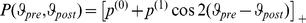
(7)Here, 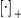 denotes rectification; i.e. 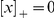 if 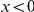, else 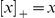. The probabilities of connection for intra-layer excitatory and inhibitory connection are identical for each of the two layers.

In order to study the scaling properties of the dynamics it is important to guarantee that the spatial mean and spatial fluctuations of the time averaged recurrent synaptic currents received by each neuron are preserved when considering networks of different sizes. This requires a suitable modification of the probabilities of connection and of the peak synaptic conductances when passing from a network of size 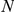 to a network of size 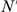 [35]. For an arbitrary peak recurrent synaptic conductance 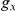, the probabilities of connection (and, correlatively the average number of pre-synaptic cells of each type) are scaled as:
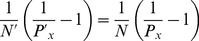
(8)and peak conductances as:
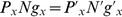
(9)Here the index 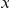 stands for different kinds of synaptic connections, each one potentially characterized by different mean probabilities of connections and connection strengths (i.e. originating from upper or lower layer excitatory or inhibitory neurons and directed toward upper or lower layer excitatory and inhibitory neurons).

Sizes between 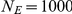 –for a total network size of 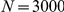 neurons– and 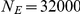 –for a total network size of 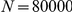 neurons– are compared in scaling analysis of synchrony properties. The parameters for 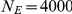 and 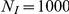 are given in Table 5. Probabilities of connection are compatible with the ranges reported by [73], [74].

**Table 5:** Probabilities of connection.

	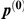	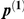	Mean target E cells	Mean target I cells
Intra-layer E to E or I	0.06	0.06	∼240	∼60
Intra-layer I to E or I	0.24	0.12	∼960	∼240
Upper E to Lower E or I	0.06	0.18	∼240	∼60
Lower E to Upper E only	0.06	0.18	∼240	—
Lower E to Upper I only	0.07	0.16	—	∼70
Inter-layer I to E or I	0.12	0.00	∼480	∼120

Probabilities of connection. The connection probability parameters *p* ^(0)^ and *p* ^(1)^ are given for a network size of *N_E_* = 4000 and *N_I_* = 1000 per layer.

### Model of the LGN input

We assume that the firing rate of a single LGN neuron, 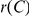 is related to the stimulus contrast, 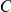, (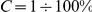) by the equation [112]:
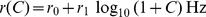
(10)where 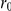 is the spontaneous activity of the neuron in dark conditions. Subsequently, we model the LGN input to a cortical cell as an AMPA-type synaptic connection with peak conductance 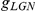, driven by homogeneous Poisson spike trains with rate 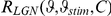,
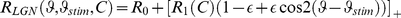
(11)with:
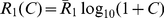
(12)Here the parameter 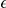 controls the broadness of tuning of the LGN input. It is set to 1 in all our simulations. Note that 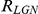 is maximum when 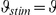. The contrast 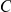 and, correspondingly, the term 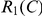 can also be time-dependent (see later section on peristimulus time histograms). The LGN input targets both layers. There is anatomical evidence that thalamo-cortical synapses target mainly layer IV and to a lesser extent layer VI [42], [45]. Accordingly, in all the simulations presented in this paper, 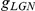 in the lower layer is smaller by a factor of two than in the upper layer. Parameters describing LGN input properties are given in Table 6.

**Table 6:** LGN input.

*gLGN*	1 nS
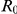	150 Hz
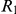	2850 Hz
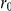	5 Hz
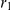	48 Hz

Parameters of the LGN input to the network *R_LGN_*(*C*). See Text and Tables S10 for more details.

For the adopted parameters, feed-forward inputs from LGN never dominate over recurrent inputs from the two layers of the network, consistently with the massively larger number of cortico-cortical synapses than thalamo-cortical synapses in the primary visual cortex [45]. The relative weight of feed-forward inputs with respect to recurrent inputs depends on contrast, doubling in our model from no more than 20% at low contrast to no more than 40% at high contrast stimulation (not shown).

An alternative parameter choice for the tuned component of the LGN input, leading to noisy input current with a larger variance, is reported in Table S1. For the noisy inputs used in this paper as well as for the ones of Table S1, the resulting sub-threshold voltage fluctuations are on the order of 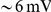 at full contrast, compatible with experimentally observed fluctuation ranges [115], [116]. Voltage fluctuations are comparable in the two regimes, because the increase in amplitude of external input current fluctuations is paralleled by a decrease in amplitude of net input conductance fluctuations, due to reduced synchrony among the recurrent inputs (see Figure S14).

More details about the mapping from stimulus contrast to input rates can be found in Text S2 and Tables S2 and S3.

### Background cortical noise

In addition to the LGN input, excitatory and inhibitory cells are driven by an untuned noisy input, representing the background firing of other cortical areas. This input is modeled by a single AMPA-type synapse per cell, with peak conductance 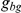 activated by Ornstein-Uhlenbeck processes [61]. Input spikes are generated independently for each cell; however all the cells share the same instantaneous input rate 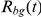 obeying the stochastic differential equation:
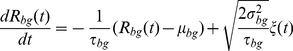
(13)where 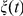 stands for Gaussian white noise and 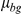 is the mean, 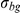 the volatility and 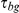 the filtering time-constant of the stochastic process. Parameters are given in Table 7.

**Table 7:** Background cortical noise.

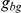	10 nS
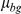	10 Hz
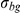	1 Hz
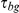	10 ms

Parameters of the background cortical input *R_bg_*.

### Numerical integration scheme

The dynamical equations are integrated using a fourth-order non-adaptive step Runge-Kutta scheme. Integration step was 0.2 ms. Because of the exponentially fast divergence of the membrane in relation with firing, particular care is needed to ensure the stability of the numerical integration of equation (1). Following [108], the numerical integration of the membrane potential 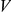 of a given neuron is stopped as soon as 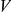 reaches a finite cutoff voltage 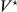. In our simulations, we use 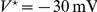. This choice ensures that the non-linear term 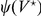 is the dominant contribution to the neuronal currents for 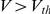. Under this condition, the leakage and the synaptic currents can be neglected, making it possible to compute analytically the time left before the actual divergence of the potential. Assuming that the integration is stopped at 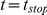 when 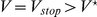, the time of the next spike is given by 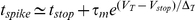. In addition, for our choice of 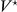, 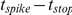 is large compared to the integration-step 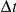, thus avoiding numerical errors in spike-time estimation due to the exponentially fast growth of 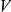 in proximity of the divergence. The membrane potential is then reset to a value 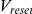 immediately after a spike.

The Ornstein-Uhlenbeck process giving 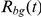 is computed using the properties of the exact solution to equation (13). This means that 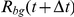 is normally distributed with mean 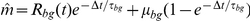 and standard deviation 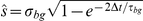 [117].

### Response tuning and contrast response function

In order to study the tuning properties of the neuronal responses we present stimuli at 12 different orientations 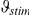 in an interval between −90 degrees and +90 degrees, at least five different contrast values 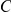 per each orientation.

Tuning curves are derived for each neuron by measuring their average firing rate for each of the tested orientations and contrasts and are characterized by computing their skewness and their circular variance [118]. The *peak rate* 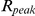 is defined as the maximum response rate generated by each single neuron over the stimulus set. Denoting by 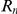 the response firing rate for a stimulus of orientation 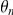, we define the complex vector:
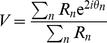
(14)The broadness of a tuning curve is quantified by *circular variance* (Mardia 1972; Ringach *et al.* 2003):
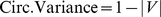
(15)where 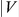 is the modulus of 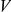. It is therefore a quantity bounded between 0 and 1. Deviation from symmetric tuning is quantified by a circular skewness coefficient (Kenney and Keeping 1962):
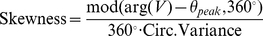
(16)where 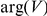 is the angle (in degrees) of the complex vector 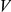 and 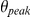 the preferred stimulus orientation. A skewness of 0 means a symmetric tuning curve and larger (or smaller) values denote tuning curves skewed toward the right (or the left). Single neuron tuning curves and the corresponding parameters for the upper layer excitatory population are shown in Figure S2. Population average tuning curves are computed after rotating single neuron tuning curves so that their maximum is at 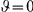 (see Figure 2A).

The contrast-response functions, CRF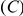, are computed for each neuron by measuring its peak firing rate (i.e. its firing response to a preferred orientation stimulus) at each given level of contrasts. Each individual CRF is fitted to a hyperbolic ratio function [60]:
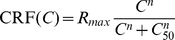
(17)Single neuron CRFs and the corresponding parameters for the upper layer excitatory population are shown in Figure S4 and population average CRFs in Figure 2B.

### Measures of synchrony

To measure the degree of macroscopic synchrony in the steady state of a network comprising an arbitrary number 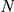 of neurons, we follow the method used in [34], [35]. It is grounded on analysis of the temporal fluctuations of macroscopic observables of the networks such as the instantaneous activity or the instantaneous membrane potential averaged over a population of neurons of size 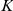. For instance, for the latter, one evaluates at a given time, 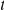, the quantity:
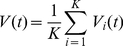
(18)The variance of the time fluctuations of 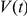 is
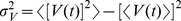
(19)where 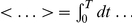 denotes time-averaging over a large time, 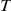. After normalization of 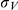 to the average over the population of the single cell membrane potentials:
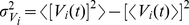
(20)one defines a *synchrony measure*, 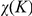 by:
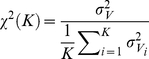
(21)This measure takes values between 0 and 1. In the limit 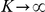 it behaves as:
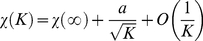
(22)where 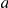 is some constant, between 0 and 1. In particular, 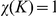, if the system is fully synchronized (i.e., 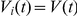 for all 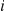), and 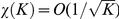 if the state of the system is asynchronous. Asynchronous and synchronous states are unambiguously characterized in the thermodynamic limit (i.e., when the number of neurons is infinite). In the asynchronous state, 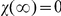. By contrast, in synchronous states, 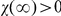.

To characterize the degree of synchrony in the membrane potentials of neurons 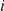 and 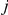, we compute the cross-correlation function:
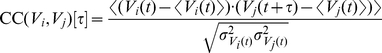
(23)The value of the normalized cross-correlogram for zero time-lag gives the pairwise crosscorrelation coefficient (CCo):
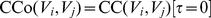
(24)To estimate the degree of synchrony in the spiking activity of these neurons, discrete spike trains are first convolved with a square window of width 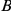, thus generating a continuous spike-count signal. Equations (23) and (24), with 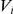 replaced by such smoothed spike trains, is used to compute crosscorrelograms and CCos for spiking activities [119]. We use a smoothing window size of 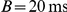.

CCos and crosscorrelograms are estimated over simulated recordings lasting 100 s of real time. For CCos between membrane potentials only pairs of neurons within a 18 region centered on an angular coordinate matching the orientation of the presented stimulus are considered. In the case of spike trains, neurons in this region whose spike train contained fewer than 100 spikes are further excluded. Various stimulus orientations are pooled together to improve the estimation of CCo distributions.

### Local field potentials

LFPs are believed to be an aggregate measure of the synaptic activity of several hundreds of neurons in the vicinity of the recording electrode [120], [121]. To evaluate the LFP in a given site, we thus arbitrarily average input synaptic currents in a small angular sector of 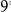 centered on the considered angular position. LFPs are estimated over neurons of the upper layer only, reflecting the fact that superficial neurons should supply the largest contribution to the signal recorded by an applied recording tip. For the normally used size of 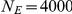 excitatory neurons and 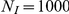 inhibitory neurons per layer, this corresponds to averaging over 200 excitatory and 50 inhibitory upper layer neurons for each considered LFP recording site.

Autocorrelograms of the LFPs are computed as:
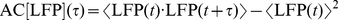
(25)the zero-lag value 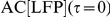 measures the variance of the temporal fluctuations of the LFP and has known size-scaling properties, which are different in synchronous and asynchronous regimes (see previous discussions and [35]).

Power spectra are computed using conventional FFT techniques, as the square modulus of the Fourier Transform of signal autocorrelation. Windowing is applied to LFP-like signal time-series to reduce unwanted frequency leakage, following the Welch method [122]). An additional moving average smoothing is applied for visualization purposes. We measure power in arbitrary logarithmic units. Since we are interested in qualitative analysis of the overall shape of the spectra rather than in absolute power estimations, for each considered regime we assign a unit value at the power at 0 Hz frequency for 0% of contrast.

Autocorrelation and spectral analysis of LFP-like signals are based on time-series lasting 100 s of real time, with a sampling rate of 5 kHz.

### Multi-unit-activity

The MUA signal reflects the spiking activity of few neurons in the immediate vicinity of the recording electrode [123]. Typically, the recorded MUA is separated in only a small number of contributing single units [124]. To evaluate MUA at a given site, following [114], we sum together the spike trains of three randomly selected cells within a small angular sector of 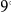 centered on the considered angular position (the same used for the evaluation of the LFP). This discrete signal is then transformed into a continuous signal by convolving it with a gaussian window (1 ms of variance).

We compute then the coherence [125] between the LFP and the MUA at a same site by taking the modulus of the normalized product of their complex Fourier Transform, using the Welch method [122], as in the case of the LFP power spectrum estimation:
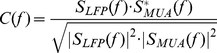
(26)where 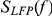 and 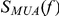 denote the (complex) Fourier transform of the autocorrelograms of the LFP and of the MUA signals, respectively. Such MUA-LFP coherence is a real quantity in the unit interval 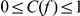, and provides an absolute (linear) measure of the phase synchronization between the two signals in different frequency bands. We average then the result over twenty different randomly chosen triplets of cells, in agreement with the experimental habit to average together different MUA recordings with only approximately similar selectivity properties [114].

### Inter-layer coupling strength and layer decoupling

Layer decoupling experiments are performed by multiplying the peak conductances of all the AMPA-type, NMDA-type and GABA-type synapses from the lower layer to the upper layer by a factor 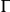 varying between 1 and 0. A value of 1 corresponds to the case of fully-interacting layers, and a value of 0 corresponds to fully uncoupled layers.

For each excitatory neuron in the upper layer, at high contrast, the peak response after layer decoupling is compared with the peak response of the same neuron in the fully coupled network case. In comparing peak responses, we take into account the fact that the tuning curves of many neurons change their preferred orientation after full or partial lower layer inactivation.

### Peristimulus time histograms

To simulate the flashing of a grating, for a given network realization we perform numerical simulations in which the tuned LGN input rate is not constant. More specifically, this tuned component is modeled according to equations (11) and (12), with a contrast modulated in time:
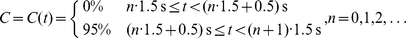
(27)Phases lasting 0.5 s in which 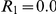 are therefore alternated with phases lasting 1 s in which 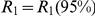, leading to a square wave time-course of the input LGN rate. We consider only cells whose preferred orientation falls within a sector 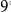 wide centered on the orientation of the presented stimulus and we use four different stimulus orientations. For each of the orientations, the stimulus is flashed 1000 times. An overall sample of 800 cells (200 per orientation) is thus considered. Spikes across stimulus repetitions and cells are binned into 2 ms bins according to their timing relative to stimulus onset. The bars in the peristimulus time histograms (PSTHs) are then evaluated as (number of spikes in a time-from-stimulus bin)/(number of stimuli repetitions)/(number of sampled cells).

### Single spike perturbation

To study the sensitivity of induced dynamics to a small perturbation, we perform a simulation in which just a single spiking even is omitted and we compare it with the unperturbed simulation. We select a putative spiking time of a neuron whose preferred orientation matches the one of the applied 95% of contrast stimulus. No spike is then sent to the post-synaptic targets, we only reset the potential and the other time-dependent parameters of the failing presynaptic neuron to their just-after-spike values. Precisely the same realizations for all the stochastic noisy input processes are taken for the unperturbed and the perturbed dynamics.

### Estimation of the largest Lyapunov exponent

We measure the largest Lyapunov exponent of the induced dynamics of the system at high contrast through a non-linear analysis of a long time-series (600 minutes of real time) of the associated LFP signal. For a thorough introduction and a rationale to the used methodologies the reader is invited to refer to textbooks like [68]. The first step is the construction of proper “embeddings” of this time-series. Given a discretely sampled time-series 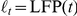, a reconstruction delay 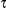 and an embedding dimension 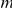, we construct a new 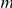-dimensional sequence:
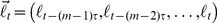
(28)It can be proven [126], [127] that the latter time-delay embedding provide in general a one-to-one image of the original phase-space attractor of the dynamics generating the measured time-series, provided that the used embedding dimension 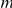 is large enough. The general idea of the method is then to identify by systematic search pairs of points 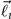 and 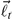 which lie at a (euclidean) distance in the delay-embedding space smaller than a specified very small 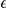. Such points are said to be *neighbors*. It is therefore possible to consider the distance 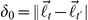 as a “small perturbation”, which should grow exponentially in time if the dynamics is chaotic. The eventual divergence of the trajectories originating by neighbor points can be monitored by the quantity 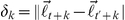. If there is a time range for which 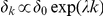 then 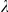 coincides with the maximum Lyapunov exponent 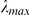 [128], [129].

We select a reconstruction delay of 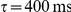, much larger than the decorrelation time of the induced LFP oscillation. The minimum embedding dimension for a consistent estimation of the largest Lyapunov exponent can be estimated by monitoring the fraction of “false neighbors” pairs, i.e. pairs of points that are neighbors in a 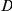-dimensional embedding (due to a projection of the attractor to a space with a too small dimensionality) but that there are no more such in an embedding with a larger dimension 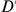 [130]. Such analysis, summarized in Figure S10, indicates a critical embedding dimension lower than five and probably larger than three (even if a precise estimation is difficult due to the presence of noise). Practically, we estimate the largest Lyapunov exponent by evaluating the quantity:
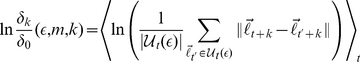
(29)for various 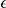, 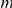 and 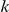, where 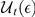 is the set of points at a distance 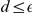 from 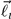 and 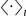 denotes average over time. If 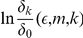 displays a linear increase in a reasonable range of 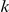 with matching slopes for different sufficiently large 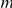 and for few decades of 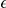, then the average slope of the linear sections of 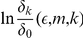 provides a robust estimation of 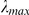. More details on our estimation of 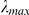 for the high contrast induced LFPs for 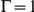 and 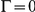 are given in the Text S1.

## Supporting Information

Figure S1 CV and firing rate distributions. Distributions of CVs and firing rates for highly active upper layer excitatory neurons (orientation preference within ±5° from stimulus) are here shown for the spontaneous activity (*C* = 0%), for the low contrast regime (*C* = 2%) and for the high contrast regime (*C* = 95%). Distributions of CVs (A–C) and of firing rates (D–F), from top to bottom, for the spontaneous activity, for the low contrast regime and for the high contrast regime.
(EPS)

Figure S2 Heterogeneity of single neuron tuning curves. A: re-centered single neuron tuning curves for 3 upper layer excitatory neurons. B: distribution of peak rates. C: distribution of tuning width. D: distribution of tuning skewness. Distributions are relative to the upper layer excitatory population.
(EPS)

Figure S3 Contrast invariance of tuning width. Tuning curves normalized to peak height, for: fully coupled layers case (A), fully uncoupled layers case (B) and strong noise case (C; see Figures S13 and S14). In cases A and B, contrast invariance is only approximate and does not hold for weak contrasts. Contrast invariance at low contrasts is improved in the strong noise case, in agreement with theory.
(EPS)

Figure S4 Heterogeneity of single neuron contrast response functions. A: single neuron CRFs for 3 upper layer excitatory neurons. B: distribution of saturation rates. C: distribution of CRF steepness. D: distribution of mid-range contrasts. Distributions are relative to the upper layer excitatory population.
(EPS)

Figure S5 Correlation coefficients for spontaneous activity. A: pairwise correlations (CCos) between membrane potentials. B: pairwise correlations between spike trains. CCos are computed as described in the Materials and Methods section of the main article.
(EPS)

Figure S6 Oscillatory structure of induced LFPs. A–B: example LFP traces from our model, evoked by a stimulus with 2% of contrast (A) or 95% of contrast (B). C–D: autocor-relograms and crosscorrelograms of evoked LFPs. For a fixed stimulus orientation, we monitor ACs and CCs of LFPs in regions responding preferentially to this stimulus orientation or to an orthogonal stimulus orientation. The analysis is performed for 2% of contrast stimuli (C) or for 95% of contrast stimuli (D). An oscillatory structure is present in LFP independently from spiking and is correlated over the entire ring. E–F: LFP temporal decorrelation at intermediate contrast levels (E: *C* = 20%; F: *C* = 4%). Damping of secondary peaks is fast at any contrast. Units are nA^2^.
(EPS)

Figure S7 Dynamics of the lower layer. A: raster plots of the activity of the lower layer excitatory neurons (lower raster) and the upper layer excitatory neurons (upper raster) in the high contrast regime dynamics (*C* = 95%). B: the latency between induced oscillations in the upper and in the lower layer is estimated through the crosscorrelogram of LFPs in the two layers (high contrast regime, *C* = 95%). The upper layer advances the lower layer ∼2∶8 ms on average.
(EPS)

Figure S8Induced responses for fully uncoupled layers: changes in tuning curves. For 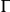 = 0 (full layer uncoupling), the dynamics of the upper layer is equivalent to the case where there is full inactivation of the lower layer. After layer uncoupling, and consistently with reference [79] (Allison and Bonds, 1994), we observe changes in preferred orientation, peak response rates and tuning curve width and skewness of single neuron tuning curves. We report here distributions of parameter changes (vertical dotted lines denote average parameters for fully coupled layers, 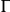 = 1). A: distribution of preferred orientation shifts. Preferred orientation of individual cells can move clockwise or anti-clockwise within a range of ∼±30° but the distribution of shifts is symmetric, with no significant change at the population level. B: distribution of peak firing rate changes. The mean peak rate change is weakly positive, reflecting the overall inhibitory nature of inter-layer coupling. C: distribution of broadness changes. On average, the width of tuning curves is slightly increased. D: distribution of skewness changes. Skewness changes are observed in both directions and their distribution is symmetric, with no significant change at the population level. In general, the large heterogeneity in the effects of layer uncoupling on tuning properties must be noted.(EPS)

Figure S9 Induced responses for fully uncoupled layers: dynamical properties. Response of the upper layer for the presentation of a 95%-contrast stimulus. A: raster plot of the excitatory population activity and associated time-histogram of the rate of spiking cells. The histogram bar heights denote the fraction of upper layer excitatory cells which _ring in the bin. Bin- size is 2 ms. B: spike trains of 6 excitatory cells highly activated by the presented stimulus. C: membrane potential traces for two neurons stimulated simultaneously at close-to-preferred orientation (2 top neurons of Panel B in red and green). This dynamics is strongly synchronous and approximately periodic. For increasing network size, oscillations tend to become more periodic, and collective synchrony does not vanish (not shown).
(EPS)

Figure S10Numerical experiments for chaos assessment. All the methods are described in Text S1. A: estimation of the minimal embedding dimension. The fraction of false neighbors is plotted against the embedding dimension for a LFP time-series generated by a full contrast preferred orientation stimulus (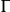 = 1, *C* = 95%). N = 1000 pairs of candidate neighbor points have been considered for each embedding dimension (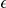
<10^−9^). A threshold of R* = 103 has been taken. A single LFP time-series long 10 hours of real time, with a sampling rate of 0.01 ms has been used for the estimation. The resulting embedding dimension appears to be *m*≥4. B–C: extraction of the largest Lyapunov exponent 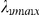 for the dynamics induced by a full contrast preferred orientation stimulus. The relative growth in time 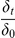 of the average separation between LFP trajectories originated from neighbor points is plotted against time, for various embedding dimensions (average over at least *N* = 1000 pairs of neighbors per considered embedding dimension). A section of exponentially fast growth (linear growth in a semilogarithmic plot, denoting deterministic chaos) is identified for sufficiently large embedding dimension in the case of a hypercolumn with interacting layers (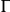 = 1, panel A), but not in the case of a hypercolumn without inter-layer interactions (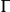 = 0, panel B).(EPS)

Figure S11 Alternative parameter choices: network with increased symmetry. We assumed in the main text that the LGN input to the lower layer is weaker. We show here results for 3 the case in which the LGN input rate to lower layer is the same as to the upper layer and in which the latency of the upper-to-lower layer connections is as short as the latency of lower-to-upper layer connections. A: raster plot of the evoked activity of the upper layer excitatory population for a 95% level of contrast stimulus. B: autocorrelogram of the evoked LFP. Units are in nA^2^. Note that synchronous chaos is still present, as evidenced by the fast damping of LFP autocorrelogram. The lower and the upper layer have now the same average firing rate and are on average in an in-phase locking.
(EPS)

Figure S12 Alternative parameter choices: network with densified inhibition. We assumed in the main text that the probability of inhibitory connection is four times larger than the probability of excitatory connections. Some Experimental studies like reference [72], however, report a probability of inhibitory connection ten times larger than for excitatory connections. We show here results for a 1∶10 ratio of excitatory to inhibitory connection probability (probabilities used are *p* ^(0)^ = 0∶6 and *p* ^(1)^ = 0.3 for intra-layer inhibitory connections and *p* ^(0)^ = 0∶3 and *p* ^(1)^ = 0∶0 for inter-layer inhibitory connections). A: raster plot of the evoked activity of the upper layer excitatory population for a 95% level of contrast stimulus. B: autocorrelogram of the evoked LFP (for different network sizes). Units are in nA^2^. Note that synchronous chaos is still present, as evidenced by the fast damping of LFP autocorrelogram, accelerating for larger network sizes. An additional effect of increased inhibitory density is a stronger tendency to resonate for low contrast stimuli. A weak “hump” at frequencies close to 45 Hz is visible even in the spectrum of spontaneous activity (not shown).
(EPS)

Figure S13 E-I connectivity is not required for the generation of oscillations. With our parameter choices, oscillations are generated thanks to delayed mutual inhibition. Excitatory neurons indeed are not required for the generation of oscillations, but are entrained by the oscillation paced by inhibitory cells. This can be proven by numerical simulations in which the activity of excitatory neurons is completely suppressed by a strong hyperpolarizing current (raster plot in panel A) or in which synapses from excitatory to inhibitory neurons are removed (raster plot in panel B). In both cases the drive to inhibitory neurons in order to maintain their rate of activity unchanged. Note that oscillations continue to exist and their frequency does not increase consistently.
(EPS)

Figure S14 Fluctuations for different noise regimes. The fluctuation level of input currents can be controlled by acting on the input rates and peak synaptic conductances. Small peak coupling conductances and large input rates yield a quasi tonic input (“small”-variance noise). Conversely, stronger peak coupling conductances and smaller input rates give rise to input currents with similar average value but stronger fluctuations in time (“large”-variance noise). The net input conductance (red = excitatory, blue = inhibitory) of an upper layer excitatory neuron driven by a full contrast stimulus is shown in panels A (small-variance noise) and C (large-variance noise). Subthreshold voltage fluctuation strength is plotted against tuned LGN input rate in panels B (small-variance noise) and D (large-variance noise). The rate ranges are different for small- and large-variance noises, but are meant to correspond conventionally in both cases to the 0%–100% contrast range (see Tables S2 and S3). For both noise regimes, the mean excitatory conductance is of ∼3–5 nS for the spontaneous activity and of ∼20 nS for full contrast stimuli and the mean inhibitory conductance is of ∼4–6 nS for the spontaneous activity and of ∼40–50 nS for full contrast stimuli. At high contrast, however, fluctuations in conductance are much stronger for small-variance input noise, because recurrent inputs are highly synchronous. Sub-threshold voltage fluctuations at high contrast are comparably strong for both noise regimes, because for small-variance noise weaker fluctuations in the input are amplified by strong conductance fluctuations. At low contrast, when the dynamics is asynchronous for both noise regimes, voltage fluctuations are stronger for large-variance noise.
(EPS)

Figure S15 High contrast dynamics for large-variance noise. Dynamics of the upper layer for the presentation of a 95%-contrast stimulus. Input noise parameters are reported in Table S1. A: raster plot of the excitatory population activity and associated time-histogram of the rate of spiking cells. The histogram bar heights denote the fraction of upper layer excitatory cells firing in the bin. Bin-size is 2 ms. B: spike trains of 6 excitatory cells highly activated by the presented stimulus. C: membrane potential traces for two neurons stimulated simultaneously at close-to-preferred orientation (2 top neurons of Panel B in red and green). This dynamics is asynchronous, as indicated by the scaling analyses of Figure S16 C–D.
(EPS)

Figure S16Temporal decorrelation and spectra of LFPs for large-variance noise. Input noise parameters are reported in Table S1. A: autocorrelogram of the LFP evoked by a high contrast stimulation. Units are nA^2^. B: power spectra of evoked LFP for various contrast levels. C: scaling with network size of the 95%-contrast synchrony factor 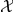. D: scaling with network size of the 95%-contrast LFP autocorrelogram. The dashed line is a power-law with exponent −0.5. This scaling is indicative of an asynchronous state. Units are nA^2^.(EPS)

Figure S17Alternative parameter choices: network with a non modulated spatial profile of inter-layer excitation. With the parameter choices assumed in the main text, the integrated effect of the inter- layer coupling is inhibitory. It is however moderately excitatory between neurons in close vertical alignment, due to the strong spatial modulation of the inter- layer excitation profile. We show here results for the case in which the spatial modulation of inter-layer excitation is removed and an equivalent average level of inter-layer excitation is used, but spread across all the angular distances (i.e. 
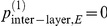
). A: raster plot of the evoked activity of the upper layer excitatory population for a 95% level of contrast stimulus. B: autocorrelogram of the evoked LFP. Note that synchronous chaos disappears, replaced by almost periodic oscillations, very similar to the case of uncoupled layers (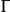 = 0, see Figure S9). Conversely, removal of inter-layer inhibition would further strengthen synchronized chaos (not shown).(EPS)

Table S1 Strong noise LGN input parameters. Parameters of the LGN input to the network for the high contrast strong noise regime. See Table S3 for more details.
(PDF)

Table S2Correspondence between C and 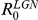 for small-variance noise. Correspondences are computed approximately, assuming that each cell receives 30 independent AMPA synaptic inputs from LGN (see Text S2). For the response of a single LGN cell we assumed 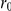 = 5 Hz and 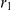 = 48 Hz.(PDF)

Table S3Correspondence between C and 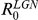 for large-variance noise. Correspondences are computed approximately, assuming that each cell receives 10 AMPA synapses from 3 independent LGN neurons (see Text S2). For the response of a single LGN cell we assumed 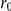 = 5 Hz and 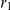 = 32 Hz.(PDF)

Text S1Detailed methods for chaos assessment. Section 1: Determination of the minimum embedding dimension. Section 2: Extraction of the largest Lyapunov exponent 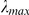.(PDF)

Text S2 Correspondence between contrast and LGN input rate. Extended description of the rationale behind the mapping between contrast and noise input parameters.
(PDF)

## References

[1] Eckhorn R, Bauer R, Jordan W, Brosch M, Kruse W (1988). Coherent oscillations: a mechanism of feature linking in the visual cortex? Multiple electrode and correlation analyses in the cat.. Biol Cybern.

[2] Gray CM, Singer W (1989). Stimulus-specific neuronal oscillations in orientation columns of cat visual cortex.. Proc Natl Acad Sci USA.

[3] Gray CM, Viana di Prisco G (1997). Stimulus-dependent neuronal oscillations and local synchronization in striate cortex of alert cat.. J Neurosci.

[4] Friedman-Hill S, Maldonado PE, Gray CM (2000). Dynamics of striate cortical activity in the alert macaque: I. Incidence and stimulus-dependence of gamma-band neuronal oscillations.. Cereb Cortex.

[5] Maldonado PE, Friedman-Hill S, Gray CM (2000). Dynamics of striate cortical activity in the alert macaque: II. Fast time scale synchronization.. Cereb Cortex.

[6] Fries P, Reynolds JH, Rorie AE, Desimone R (2001). Modulation of oscillatory neuronal synchronization by selective visual attention.. Science.

[7] Logothetis NK, Pauls J, Augath M, Trinath T, Oeltermann A (2001). Neurophysiological investigation of the basis of the fMRI signal.. Nature.

[8] Samonds JM, Bonds AB (2004). Gamma oscillation maintains stimulus structure-dependent synchronization in cat visual cortex.. J Neurophysiol.

[9] Henrie JA, Shapley R (2005). LFP power spectra in V1 cortex: the graded effect of stimulus contrast.. J Neurophysiol.

[10] Belitski A, Gretton A, Magri C, Murayama Y, Montemurro MA (2008). Low-frequency local field potentials and spikes in primary visual cortex convey independent visual information.. J Neurosci.

[11] Fries P, Womelsdorf T, Oostenveld R, Desimone R (2008). The effects of visual stimulation and selective visual attention on rhythmic neuronal synchronization in macaque area V4.. J Neurosci.

[12] Gieselmann MA, Thiele A (2008). Comparison of spatial integration and surround suppression characteristics in spiking activity and the local field potential in macaque V1.. Eur J Neurosci.

[13] Zhou Z, Bernard MR, Bonds AB (2008). Deconstruction of spatial integrity in visual stimulus detected by modulation of synchronized activity in cat visual cortex.. J Neurosci.

[14] Lima B, Singer W, Chen NH, Neuenschwander S (2010). Synchronization Dynamics in Response to Plaid Stimuli in Monkey V1.. Cereb Cortex.

[15] Tallon-Baudry C, Bertrand O, Delpuech C, Pernier J (1996). Stimulus specificity of phase-locked and non-phase-locked 40 Hz visual responses in human.. J Neurosci.

[16] Rols G, Tallon-Baudry C, Girard P, Bertrand O, Bullier J (2001). Cortical mapping of gamma oscillations in areas V1 and V4 of the macaque monkey.. Vis Neurosci.

[17] Kreiter AK, Singer W (1996). Stimulus-dependent synchronization of neuronal responses in the visual cortex of the awake macaque monkey.. J Neurosci.

[18] Ray S, Maunsell JH (2010). Differences in gamma frequencies across visual cortex restrict their possible use in computation.. Neuron.

[19] Nowak LG, Bullier J, Miller R (2000). Cross correlograms for neuronal spike trains. Different types of temporal correlation in neocortex, their origin and significance.. Time and the Brain, Conceptual advances in Brain Research.

[20] Berens P, Keliris GA, Ecker AS, Logothetis NK, Tolias AS (2008). Feature selectivity of the gamma-band of the local field potential in primate primary visual cortex.. Front Neurosci.

[21] Whittington MA, Traub RD, Jefferys JG (1995). Synchronized oscillations in interneuron networks driven by metabotropic glutamate receptor activation.. Nature.

[22] Bartos M, Vida I, Jonas P (2007). Synaptic mechanisms of synchronized gamma oscillations in inhibitory interneuron networks.. Nat Rev Neurosci.

[23] Cardin JA, Carlén M, Meletis K, Knoblich U, Zhang F (2009). Driving fast-spiking cells induces gamma rhythm and controls sensory responses.. Nature.

[24] Sohal VS, Zhang F, Yizhar O, Deisseroth K (2009). Parvalbumin neurons and gamma rhythms enhance cortical circuit performance.. Nature.

[25] Brunel N, Hakim V (1999). Fast global oscillations in networks of integrate-and-fire neurons with low firing rates.. Neural Comput.

[26] Whittington MA, Traub RD, Kopell N, Ermentrout GB, Buhl EH (2000). Inhibition-based rhythms: Experimental and mathematical observation on network dynamics.. Int J Psychophysiol.

[27] Brunel N, Wang XJ (2003). What determines the frequency of fast network oscillations with irregular neural discharges?. J Neurophysiol.

[28] Brunel N, Hansel D (2006). How noise affects the synchronization properties of recurrent networks of inhibitory neurons.. Neural Comput.

[29] Brunel N, Hakim V (2008). Sparsely synchronized neuronal oscillations.. Chaos.

[30] Wang XJ (2010). Neurophysiological and Computational Principles of Cortical Rhythms in Cognition.. Physiol Rev.

[31] Kayser C, König P (2004). Stimulus locking and feature selectivity prevail in complementary frequency ranges of V1 local field potentials.. Eur J Neurosci.

[32] Nir Y, Fisch L, Mukamel R, Gelbard-Sagiv H, Arieli A (2007). Coupling between neuronal firing rate, gamma LFP, and BOLD fMRI is related to interneuronal correlations.. Curr Bio.

[33] Briggs F, Usrey WM (2009). Modulation of gamma-band activity across local cortical circuits.. Front Integr Neurosci.

[34] Hansel D, Sompolinsky H (1996). Chaos and synchrony in a model of a hypercolumn in visual cortex.. J Comput Neurosci.

[35] Golomb D, Hansel D (2000). The number of synaptic inputs and the synchrony of large, sparse neuronal networks.. Neural Comp.

[36] Rennie CJ, Wright JJ, Robinson PA (2000). Mechanisms of Cortical Electrical Activity and Emergence of Gamma Rhythm.. J Theor Bio.

[37] Mazzoni A, Panzeri S, Logothetis NK, Brunel N (2008). Encoding of naturalistic stimuli by local field potential spectra in networks of excitatory and inhibitory neurons.. PLOS Comput Bio.

[38] Kang K, Shelley MJ, Henrie JA, Shapley RM (2010). LFP spectral peaks in V1 cortex: network resonance and cortico-cortical feedback.. J Comput Neurosci.

[39] Roxin A, Brunel N, Hansel D (2005). The role of delays in shaping the spatio-temporal dynamics of neuronal activity in large networks.. Phys Rev Lett.

[40] Roxin A, Brunel N, Hansel D (2006). Rate Models with Delays and the Dynamics of Large Networks of Spiking Neurons.. Progr Theor Phys.

[41] Battaglia D, Brunel N, Hansel D (2007). Temporal decorrelation of collective oscillations in neural networks with local inhibition and long-range excitation.. Phys Rev Lett.

[42] Blasdel GG, Lund JS (1983). Termination of afferent axons in macaque striate cortex.. J Neurosci.

[43] Ferster D, Chung S, Wheat H (1996). Orientation selectivity of thalamic input to simple cells of cat visual cortex.. Nature.

[44] Callaway EM (1998). Local circuits in primary visual cortex of the macaque monkey.. Annu Rev Neurosci.

[45] Binzegger T, Douglas RJ, Martin KAC (2004). A quantitative map of the circuit of cat primary visual cortex.. J Neurosci.

[46] Sincich LC, Horton JC (2005). The circuitry of V1 and V2: integration of color, form, and motion.. Annu Rev Neurosci.

[47] Briggs F (2010). Organizing principles of cortical layer 6.. Front Neural Circuits.

[48] Thomson AM (2010). Neocortical layer 6, a review.. Front Neuroanat.

[49] Raizada R, Grossberg S (2003). Towards a theory of the laminar architecture of cerebral cortex: computational clues from the visual system.. Cereb Cortex.

[50] Thomson AM, Bannister AP (2003). Interlaminar connections in the neocortex.. Cereb Cortex.

[51] Hirsch JA, Martinez LM (2006). Laminar processing in the visual cortical column.. Curr Op Neurobio.

[52] Stepanyants A, Martinez LM, Ferecskó AS, Kisvárday ZF (2009). The fractions of short- and long-range connections in the visual cortex.. Proc Natl Acad Sci USA.

[53] Ben-Yishai R, Bar-Or RL, Sompolinsky H (1995). Theory of orientation tuning in visual cortex.. Proc Natl Acad Sci USA.

[54] Hansel D, Sompolinsky H (1998). Modeling Feature Selectivity in Local Cortical Circuits, in Methods in Neuronal Modeling: From Synapse to Networks, Koch C and Segev I editors, Chapter 13, 2nd edition.

[55] Hubel DH, Wiesel TN (1962). Receptive fields, binocular interaction and functional architecture in the cat's visual cortex.. J Physiol.

[56] Ahmed B, Anderson JC, Martin KAC, Nelson JC (1997). Map of the synapses onto layer 4 basket cells of the primary visual cortex of the cat.. J Comp Neurol.

[57] Anderson JS, Lampl I, Gillespie DC, Ferster D (2000). The contribution of noise to contrast invariance of orientation tuning in cat visual cortex.. Science.

[58] Hansel D, van Vreeswijk C (2002). How noise contributes to contrast invariance of orientation tuning in cat visual cortex.. J Neurosci.

[59] Persi E, Hansel D, Nowak L, Barone P, van Vreeswijk C (2010). Power-law input-output transfer functions explain the contrast response and tuning properties of neurons in visual cortex.. PLOS Comput Bio.

[60] Contreras D, Palmer L (2003). Response to contrast of electrophysiologically defined cell classes in primary visual cortex.. J Neurosci.

[61] Rudolph M, Destexhe A (2004). Inferring network activity from synaptic noise.. J Physiol Paris.

[62] Womelsdorf T, Schoffelen JM, Oostenveld R, Singer W, Desimone R (2007). Modulation of neuronal interactions through neuronal synchronization.. Science.

[63] Burns SP, Xing D, Shelley MJ, Shapley RM (2010). Searching for temporal phase coherence in the cortical network with a time-frequency analysis of the local field potential.. J Neurosci.

[64] Wallace MN, Palmer AR (2008). Laminar differences in the response properties of cells in the primary auditory cortex.. Exp Brain Res.

[65] Sakata S, Harris KD (2009). Laminar Structure of Spontaneous and Sensory-Evoked Population Activity in Auditory Cortex.. Neuron.

[66] Schuster HG, Just W (2005). Deterministic chaos..

[67] Newhouse S, Ruelle D, Takens F (1978). Occurrence of strange axiom A attractors near quasi periodic flows on <$>\scale 50%\raster="rg1"<$>, *m*≥3.. Commun Math Phys.

[68] Kantz H, Schreiber T (2004). Nonlinear time-series analysis, 2nd edition..

[69] Katz LC (1987). Local circuitry of identified projection neurons in cat visual cortex brain slices.. J Neurosci.

[70] Gilbert CD (1977). Laminar differences in receptive field properties of cells in cat primary visual cortex.. J Physiol.

[71] Martinez LM, Wang Q, Reid R, Pillai C, Alonso JM (2005). Receptive field structure varies with layer in the primary visual cortex.. Nat Neurosci.

[72] Holmgren C, Harkany T, Svennenfors B, Zilberter Y (2003). Pyramidal cell communication within local networks in layer 2/3 of rat neocortex.. J Physiol.

[73] Thomson AM, West D, Wang Y, Bannister AP (2002). Synaptic connections and small circuits involving excitatory and inhibitory neurons in layers 2–5 of adult rat and cat neocortex: triple intracellular recordings and biocytin labelling in vitro.. Cereb Cortex.

[74] Yoshimura Y, Callaway EM (2005). Fine-scale specificity of cortical networks depends on inhibitory cell type and connectivity.. Nat Neurosci.

[75] Brunel N (2000). Dynamics of sparsely connected networks of excitatory and inhibitory spiking neurons.. J Comput Neurosci.

[76] Hansel D, Mato G (2003). Asynchronous states and the emergence of synchrony in large networks of interacting excitatory and inhibitory neurons.. Neural Comput.

[77] Geisler C, Brunel N, Wang XJ (2005). Contribution of intrinsic membrane dynamics to fast network oscillations with irregular neuronal discharges.. J Neurophysiol.

[78] Horton JC, Adams DL (2005). The cortical column: a structure without a function.. Philos Trans R Soc Lond B Biol Sci.

[79] Allison JD, Bonds AB (1994). Inactivation of the infragranular striate cortex broadens orientation tuning of supragranular visual neurons in the cat.. Exp Brain Res.

[80] Yu J, Ferster D (2010). Membrane potential synchrony in primary visual cortex during sensory stimulation.. Neuron.

[81] Toyama K, Kimura M, Tanaka K (1981). Organization of cat visual cortex as investigated by cross-correlation technique.. J Neurophysiol.

[82] Schwarz C, Bolz J (1991). Functional specificity of a long-range horizontal connection in cat visual cortex: a cross-correlation study.. J Neurosci.

[83] Hata Y, Tsumoto T, Sato H, Tamura H (1991). Horizontal interactions between cortical neurones studied by cross-correlation analysis in the cat.. J Physiol.

[84] Ghose GM, Freeman RD, Ohzawa I (1994). Local intracortical connections in the cat's visual cortex: postnatal development and plasticity.. J Neurophysiol.

[85] Das A, Gilbert CD (1995). Receptive field expansion in adult visual cortex in linked to dynamic changes in strength of cortical connections.. J Neurophysiol.

[86] Montani F, Kohn A, Smith MA, Schultz SR (2007). The role of correlations in direction and contrast coding in the primary visual cortex.. J Neurosci.

[87] Tao L, Shelley M, McLaughlin D, Shapley R (2004). An egalitarian network model for the emergence of simple and complex cells in visual cortex.. Proc Natl Acad Sci USA.

[88] Tao L, Cai D, McLaughlin D, Shelley MJ, Shapley R (2006). Orientation selectivity in visual cortex by fluctuation-controlled criticality.. Proc Natl Acad Sci USA.

[89] Sompolinsky H, Crisanti A, Sommers HJ (1988). Chaos in random neural networks.. Phys Rev Lett.

[90] Hansel D, Sompolinsky H (1993). Solvable model of spatiotemporal chaos.. Phys Rev Lett.

[91] Van Vreeswijk C, Sompolinsky H (1996). Chaos in neuronal networks with balanced excitatory and inhibitory activity.. Science.

[92] Van Vreeswijk C, Sompolinsky H (1998). Chaotic Balanced State in a Model of Cortical Circuits.. Neural Comput.

[93] Marre O, Yger P, Davison AP, Frégnac Y (2009). Reliable recall of spontaneous activity patterns in cortical networks.. J Neurosci.

[94] Zillmer R, Brunel N, Hansel D (2009). Very long transients, irregular firing, and chaotic dynamics in networks of randomly connected inhibitory integrate-and-fire neurons.. Phys Rev E.

[95] Jahnke S, Memmesheimer RM, Timme M (2009). How chaotic is the balanced state?. Front Comp Neurosci.

[96] Sussillo D, Abbott LF (2009). Generating coherent patterns of activity from chaotic neural networks.. Neuron.

[97] Rajan K, Abbott LF, Sompolinsky H (2010). Stimulus-dependent suppression of chaos in recurrent neural networks.. Phys Rev E.

[98] Shibata T, Kaneko K (1998). Collective Chaos.. Phys Rev Lett.

[99] Pecora L, Carroll T (1990). Synchronization in chaotic systems.. Phys Rev Lett.

[100] Kaneko K, Tsuda I (2001). Complex systems: chaos and beyond. A constructive approach with applications in life sciences..

[101] Hansel D, Sompolinsky H (1992). Synchronization and computation in a chaotic neural network.. Phys Rev Lett.

[102] Bolz J, Gilbert CD (1986). Generation of end-inhibition in the visual cortex via interlaminar connections.. Nature.

[103] Schwark HD, Malpeli JG, Weyand TG, Lee C (1986). Cat area 17. II. Response properties of infragranular layer neurons in the absence of supragranular layer activity.. J Neurophysiol.

[104] London M, Roth A, Beeren L, Hausser M, Latham PE (2010). Sensitivity to perturbations in vivo implies high noise and suggests rate coding in cortex.. Nature.

[105] Wilson HR, Cowan JD (1972). Excitatory and inhibitory interactions in localized populations of model neurons.. Biophys J.

[106] Li Z, Hopfield JJ (1989). Modeling the olfactory bulb and its neural oscillatory processings.. Biol Cybern.

[107] Li Z, Dayan P (1999). Computational differences between asymmetrical and symmetrical networks.. Network.

[108] Fourcaud-Trocmé N, Hansel D, van Vreeswijk C, Brunel N (2003). How spike generation mechanisms determine the neuronal response to fluctuating inputs.. J Neurosci.

[109] Badel L, Lefort S, Brette R, Petersen CC, Gerstner W (2008). Dynamic I–V curves are reliable predictors of naturalistic pyramidal-neuron voltage traces.. J Neurophysiol.

[110] McCormick DA, Connors BW, Lighthall JW, Prince DA (1985). Comparative electrophysiology of pyramidal and sparsely spiny stellate neurons of the neocortex.. J Neurophysiol.

[111] Badel L, Lefort S, Berger TK, Petersen CC, Gerstner W (2008). Extracting non-linear integrate-and-fire models from experimental data using dynamic I–V curves.. Biol Cybern.

[112] Somers DC, Nelson SB, Sur M (1995). An emergent model of orientation selectivity in cat visual cortical simple cells.. J Neurosci.

[113] Ardid S, Wang XJ, Compte A (2007). An integrated microcircuit model of attentional processing in the neocortex.. J Neurosci.

[114] Ardid S, Wang XJ, Gomez-Cabrero D, Compte A (2010). Reconciling coherent oscillation with modulation of irregular spiking activity in selective attention: gamma-range synchronization between sensory and executive cortical areas.. J Neurosci.

[115] Anderson JS, Carandini M, Ferster D (2000). Orientation tuning of input conductance, excitation, and inhibition in cat primary visual cortex.. J Neurophysiol.

[116] Cardin JA, Palmer LA, Contreras D (2007). Stimulus feature selectivity in excitatory and inhibitory neurons in primary visual cortex.. J Neurosci.

[117] Gillespie DT (1996). Exact numerical simulation of the Ornstein?Uhlenbeck process and its integral.. Phys Rev E.

[118] Ringach DL, Shapley RM, Hawken MJ (2002). Orientation selectivity in macaque V1: diversity and laminar dependence.. J Neurosci.

[119] Renart A, de la Rocha J, Bartho P, Hollender L, Parga N (2010). The asynchronous state in cortical circuits.. Science.

[120] Mitzdorf U (1985). Current source-density method and application in cat cerebral cortex: investigation of evoked potentials and eeg phenomena.. Physiol Rev.

[121] Katzner S, Nauhaus I, Benucci A, Bonin V, Ringach RL (2009). Local origin of field potentials in visual cortex.. Neuron.

[122] Oppenheim AV, Schafer RW, Buck JA (1999). Discrete-time signal processing..

[123] Buzsáki G (2004). Large-scale recording of neuronal ensembles.. Nat Neurosci.

[124] Quiroga QR, Nadasdy Z, Ben-Shaul Y (2004). Unsupervised spike detection and sorting with wavelets and superparamagnetic clustering.. Neural Comp.

[125] Mitra PP, Pesaran B (1999). Analysis of dynamic brain imaging data.. Biophys J.

[126] Takens F (1981). Detecting strange attractors in turbulence..

[127] Sauer T, Yorke J, Casdagli M (1991). Embedology.. J Stat Phys.

[128] Rosenstein MT, Collins JJ, De Luca CJ (1993). A practical method for calculating largest Lyapunov exponents from small data set.. Physica D.

[129] Kantz H (1994). A robust method to estimate the maximal Lyapunov exponent of a time series.. Phys Lett A.

[130] Kennel MB, Brown R, Abarbanel HDI (1992). Determining embedding dimensions for phase-space reconstruction using a geometrical construction.. Phys Rev A.

