# Supplementary material for: Synchronous Chaos and Broad Band Gamma Rhythm in a Minimal Multi-Layer Model of Primary Visual Cortex
Source: PLoS Comput Biol. 2011 Oct 6;7(10):e1002176. doi: 10.1371/journal.pcbi.1002176 (PMC3188510; doi:10.1371/journal.pcbi.1002176)
Supplement: Table S1 — Strong noise LGN input parameters. Parameters of the LGN input to the network for the high contrast strong noise regime. See Table S3 for more details. (PDF) [file pcbi.1002176.s018.pdf]

g_{LGN} (nS)	R_0 (Hz)	R_1 (Hz)	r_0 (Hz)	r_1 (Hz)
10	15	190	5	31

Table S1. Strong noise LGN input parameters. Parameters of the LGN input to the network for the high contrast strong noise regime. See Table S3 for more details.
