# Supplementary material for: Synchronous Chaos and Broad Band Gamma Rhythm in a Minimal Multi-Layer Model of Primary Visual Cortex
Source: PLoS Comput Biol. 2011 Oct 6;7(10):e1002176. doi: 10.1371/journal.pcbi.1002176 (PMC3188510; doi:10.1371/journal.pcbi.1002176)
Supplement: Table S2 — Correspondence between C and for small-variance noise. Correspondences are computed approximately, assuming that each cell receives 30 independent AMPA synaptic inputs from LGN (see Text S2). For the response of a single LGN cell we assumed = 5 Hz and = 48 Hz. (PDF) [file pcbi.1002176.s019.pdf]

C	0%	1%	2%	4%	20%	95%
R_0 (Hz)	150					
R_1 (Hz)	0	430	690	1000	1900	2850
g_{LGN} (nS)	1					

Table S2. Correspondence between C and R_0^{LGN} for weak noise. Correspondences are computed approximately, assuming that each cell receives 30 independent AMPA synaptic inputs from LGN. For the response of a single LGN cell we assumed $r_0 = 5$ Hz and $r_1 = 48$ Hz.
