# Supplementary material for: Synchronous Chaos and Broad Band Gamma Rhythm in a Minimal Multi-Layer Model of Primary Visual Cortex
Source: PLoS Comput Biol. 2011 Oct 6;7(10):e1002176. doi: 10.1371/journal.pcbi.1002176 (PMC3188510; doi:10.1371/journal.pcbi.1002176)
Supplement: Table S3 — Correspondence between C and for large-variance noise. Correspondences are computed approximately, assuming that each cell receives 10 AMPA synapses from 3 independent LGN neurons (see Text S2). For the response of a single LGN cell we assumed = 5 Hz and = 32 Hz. (PDF) [file pcbi.1002176.s020.pdf]

C	0%	1%	3%	9%	30%	95%
R_0 (Hz)	15					
R_1 (Hz)	0	30	60	100	140	190
g_{LGN} (nS)	10					

Table S3. Correspondence between C and R_0^{LGN} for strong noise. Correspondences are computed approximately, assuming that each cell receives 10 AMPA synapses from 3 independent LGN neurons. For the response of a single LGN cell we assumed $r_0 = 5$ Hz and $r_1 = 32$ Hz.
