# Supplementary material for: Synchronous Chaos and Broad Band Gamma Rhythm in a Minimal Multi-Layer Model of Primary Visual Cortex
Source: PLoS Comput Biol. 2011 Oct 6;7(10):e1002176. doi: 10.1371/journal.pcbi.1002176 (PMC3188510; doi:10.1371/journal.pcbi.1002176)
Supplement: Text S1 — Detailed methods for chaos assessment. Section 1: Determination of the minimum embedding dimension. Section 2: Extraction of the largest Lyapunov exponent . (PDF) [file pcbi.1002176.s021.pdf]

Text S10. Detailed methods for chaos assessment

1 Determination of the minimum embedding dimension

A time-delay embedding of a (sufficiently long) time-series generated by a dynamical system provides a one-to-one image of the attractor in phase-space of the dynamical system itself, for any embedding dimension larger than a minimum m (which can be larger than the fractal dimension of the attractor to reconstruct, in presence of noisy timeseries)[Ref i]. A method to estimate this minimum embedding dimension (for a fixed reconstruction delay) is the method of false neighbors [Ref ii]. If a D-dimensional attractor is projected to a space with a smaller dimensionality, some spurious self-intersections can be generated and give rise thus to false neighbors, which will be no more found to be neighbors for any embedding with a sufficiently larger dimensionality. When considering the divergence of the trajectories originated by a pair of false neighbor points over very short time ranges (e.g. one sampling step), this divergence will be found to be orders of magnitude faster than the typical divergence rate between true neighbor points. An empiric criterion to identify false neighbors is therefore to compute the quantity:

$$R_t = \frac{\|\vec{\ell}_{t+1} - \vec{\ell}_{t'+1}\|}{\|\vec{\ell}_t - \vec{\ell}_{t'}\|}$$

for any pair of candidate neighbor points $\vec{\ell}_t$ and $\vec{\ell}_{t'}$. Outlier pairs with an anomalously high rate of initial divergence R_t larger than a large heuristic threshold R^* are identified as potential false neighbors. The fraction of potential false neighbors is then plotted for different embeddings against the embedding dimension m (see Figure S11A). The minimal embedding dimension is given by the minimum dimension for which the fraction of false neighbors has fallen very close to zero. In the case of LFP time-series evoked by full contrast stimuli a safe choice for the minimum embedding dimension appears to be $m = 4$.

2 Extraction of λ_{max}

We monitor then the growth of the quantity $\frac{\delta_t}{\delta_0}(\epsilon, m, t)$ for different embedding dimensions m and ball diameters ϵ. We compute averages over at least $N = 1000$ pairs of true neighbor points per embedding dimension, including only pairs separated temporally

by a minimum window of $\tau_{sep} > 800$ ms (twice the reconstruction delay used for the embedding), to safely avoid correlated pairs. The divergence of trajectories originated from neighboring points is very fast and reaches very quickly a saturation level, due to the finite volume of the attractor (Figure S10-2). In the case of the LFP evoked by a preferred-orientation stimulus at full contrast (coupled layers, $\Gamma = 1$), we can identify a region lasting several ms in which this divergence is exponentially fast, as indicated by a section of exponential growth (linear in a semilogarithmic plot) of the quantity $\frac{\delta_t}{\delta_0}$ (see Figure S11-B). Linear interpolation in the range 0.5 ms $<$ t $<$ 3.5 ms provides an estimate of $\lambda_{max} = 2.2 \pm 0.6$ ms^{-1}, robust in a range of $10^{-11} < \epsilon < 10^{-9}$ (three decades of perturbation strengths) and for embedding dimension $m \geq 4$.

Using exactly the same parameters for the non-linear analysis of time-series of LFP evoked by a preferred-orientation stimulus at full contrast with uncoupled layers ($\Gamma = 0$), we fail in identifying any time interval of exponentially fast growth of $\frac{\delta_t}{\delta_0}$ (Figure S11-C). In this case, the divergence is still fast, although slower than for coupled layers, however it is due to noise and not intrinsically to deterministic chaos. Therefore, chaos disappears when the inter-layer interactions are suppressed.

References

[Ref i] Kantz H, Schreiber T (2004) Nonlinear time-series analysis, 2nd edition. Cambridge University Press.

[Ref ii] Kennel MB, Brown R, Abarbanel HDI (1992) Determining embedding dimensions for phase-space reconstruction using a geometrical construction. Phys Rev A 45: 3403.

[Ref iii] Kantz H (1994) A robust method to estimate the maximal Lyapunov exponent of a time series. Phys Lett A 185:77.
