# Supplementary material for: Synchronous Chaos and Broad Band Gamma Rhythm in a Minimal Multi-Layer Model of Primary Visual Cortex
Source: PLoS Comput Biol. 2011 Oct 6;7(10):e1002176. doi: 10.1371/journal.pcbi.1002176 (PMC3188510; doi:10.1371/journal.pcbi.1002176)
Supplement: Text S2 — Correspondence between contrast and LGN input rate. Extended description of the rationale behind the mapping between contrast and noise input parameters. (PDF) [file pcbi.1002176.s022.pdf]

S20. Correspondence between contrast and LGN input rate

To a specific contrast C of the stimulus corresponds a value of the firing rate R_{LGN} of the input Poisson processes representing sensory-evoked thalamocortical inputs. The LGN input rate to a cell at coordinate ϑ, for a stimulus of orientation ϑ_{stim} and contrast C , is given by:

$$R_{LGN}(\vartheta, \vartheta_{stim}, C) = R_0 + [R_1(C)(1 - \epsilon + \epsilon \cos 2(\vartheta - \vartheta_{stim})]_+$$

Here we discuss the rationale for our choice of the parameters R_0 and $R_1(C)$. The response rate of a single LGN cell to a stimulus of contrast C can be parametrized by the following relation:

$$r(C) = r_0 + r_1 \log_{10}(1 + C)$$

where the contrast varies in the range $C = 0 \div 100\%$ [Ref i, Ref ii]. Each neuron in the upper layer is assumed to receive inputs from N_{cells} LGN cells, each one establishing N_{syn} AMPA synapses on the target cell. In absence of a visual stimulus ($C = 0\%$) the total LGN input rate R_0 to a cell in the upper layer at any angular position ϑ is given by:

$$R_{LGN}(\vartheta, \vartheta_{stim}, 0\%) = R_0 = N_{cells} r_0$$

When the stimulus orientation ϑ_{stim} corresponding to the cell angular coordinate and when $C = 100\%$, the cell receives the maximum possible input from LGN, given by:

$$R_{LGN}(\vartheta_{stim}, \vartheta_{stim}, 100\%) = R_0 + R_1 \simeq N_{cells}(r_0 + 2r_1)$$

When specific values for r_0, r_1, N_{syn} and N_{cells} are set, the two previous equations allow us to derive R_0 and $R_1(C)$. The peak conductance g_{LGN} is then selected according to the following formula:

$$g_{LGN} = N_{syn}\, g_{AMPA}$$

where g_{AMPA} corresponds to the strength of an individual AMPA recurrent connection. This peak conductance is divided by a factor 2 for LGN inputs toward the lower layer.

The exact correspondence between C and R_{LGN} for the contrast values used in our simulations is presented in Table S21 (small-variance noise regime, see main paper) and in Table S22 (large-variance noise regime, see Figures S15, S16 and S17).

References

[Ref i] Chino YM, Kaplan E (1988) Abnormal orientation bias of LGN neurons in strabismic cats. Invest Ophthalmol Vis Sci 29:644–648.

[Ref ii] Somers DC, Nelson SB, Sur M (1995) An emergent model of orientation selectivity in cat visual cortical simple cells. J. Neurosci. 15:5448–5465.
